# Supplementary material for: GARP and EARP are required for efficient BoHV-1 replication as identified by a genome wide CRISPR knockout screen
Source: PLoS Pathog. 2023 Dec 6;19(12):e1011822. doi: 10.1371/journal.ppat.1011822 (PMC10727446; doi:10.1371/journal.ppat.1011822)
Supplement: S1 Text — (DOCX) [file ppat.1011822.s008.docx]

**GARP and EARP are required for efficient BoHV-1 replication as identified by a genome wide CRISPR knockout screen**

Wenfang S. Tan^1,*^, Enguang Rong^1,^ ^†^ ^[[1]](#footnote-1)^, Inga Dry^1^, Simon G. Lillico^2,4^, Andy Law^3^, Paul Digard^1^, Bruce Whitelaw^2,4^, Robert G. Dalziel^1^

1. Division of Infection and Immunity, University of Edinburgh, Edinburgh, Scotland, United Kingdom

2. Division of Functional Genetics and Development, University of Edinburgh, Edinburgh, Scotland, United Kingdom

3. Division of Genetics and Genomics, University of Edinburgh, Edinburgh, Scotland, United Kingdom

4. Centre for Tropical Livestock Genetics and Health, the Roslin Institute, Easter Bush Campus, University of Edinburgh, , Edinburgh, Scotland, United Kingdom

* [wtan2@exseed.ed.ac.uk](mailto:wtan2@exseed.ed.ac.uk)

**Keywords:** CRISPR/Cas9, knockout screen, cattle, BoHV-1, GARP, EARP

**Supplementary materials and methods**

**References**

Table of Contents

[Genome-wide library design 3](#_Toc152082709)

[Library cloning 3](#_Toc152082710)

[Focused CRISPR library design and cloning 4](#_Toc152082711)

[CRISPRi guide RNA design and cloning 4](#_Toc152082712)

[Tissue culture 4](#_Toc152082713)

[Transfection 4](#_Toc152082714)

[Serum free lentivirus library packaging, titration and transduction 5](#_Toc152082715)

[TALEN assembly and in vitro transcription of TALEN mRNAs 5](#_Toc152082716)

[Individual sgRNA cloning and in vitro transcription of sgRNAs 5](#_Toc152082717)

[Gene editing efficiency testing by T7E1 digestion and TIDE analysis 6](#_Toc152082718)

[Generating the Cas9+/+, and Cas9+/+; TRIM5 -/- clones 6](#_Toc152082719)

[Colony isolation by dilutional cloning and genotyping 6](#_Toc152082720)

[Production of rescue cells 6](#_Toc152082721)

[BoHV-1 infection 6](#_Toc152082722)

[Plaque assays 7](#_Toc152082723)

[FACS sort 7](#_Toc152082724)

[genomic DNA isolation from MDBK cells and PCR for NextSeq 7](#_Toc152082725)

[NextSeq reads processing and data analysis 8](#_Toc152082726)

[Gene Ontology analysis 8](#_Toc152082727)

[STRING clustering analysis 8](#_Toc152082728)

[Viral genomic DNA sample collection, quantification and genome to pfu growth curves 8](#_Toc152082729)

[Reverse transcription, Quantitative PCR of viral transcripts and viral mRNA/viral DNA growth curves 8](#_Toc152082730)

[VP26-GFP growth tracing 9](#_Toc152082731)

[Cloning and transfection of constructs expressing candidate proteins of BoHV-1 to identify the FLI antibody target 9](#_Toc152082732)

[Concentration of viruses and western blot staining for VP8 to VP26-GFP ratios 9](#_Toc152082733)

[Coomassie staining and Mass spec sample preparation 10](#_Toc152082734)

[Immunofluorescent staining and confocal imaging 10](#_Toc152082735)

[Statistical analysis 10](#_Toc152082736)

[Table A. Plasmids used in the study 11](#_Toc152082737)

[Table B. Individual CRISPRs and TALENs used in this study 12](#_Toc152082738)

[Table C. Oligos used to clone CRISPR3i vectors for gene knockdown 13](#_Toc152082739)

[Table D. Primers used for cloning, gene targeting and qPCR 15](#_Toc152082740)

[Table E. Primers used for NextSeq 16](#_Toc152082741)

[Table F. Reagents and consumables used in the study 17](#_Toc152082742)

[References 19](#_Toc152082743)

# Genome-wide library design

**Step 1**: **Extract common coding sequences among CDS isoforms**

The latest RefSeq annotations for the bovine genome assemblies (as of Nov. 2017) GCF_000003055.6 from UMD3.1.1 and GCF_000003205.7 from btau5.01 were downloaded from the FTP of NCBI, and the Y chromosome from btau5.01 was combined with UMD3.1.1. For each protein coding gene, a python script utilizing the “intersect” sub-command from the bedtools v2.26.0 suite was used to extract coordinates of shared coding sequences among CDS isoforms.

**Step 2: Extract candidate CRISPRs**

These coordinates were then used to extract genomic sequences from the assembly using the bedtools v2.26.0 “getfasta” sub-command. All 20bp sequences immediately upstream of “NGG” trinucleotides on both the “+” and “-” strands were extracted but only guides that met the following criteria were included in the candidate list: **a**. have a 20-80% GC content; **b**. do not contain BbsI binding sites “GAAGAC” and “GTCTTC”, and **c**. do not contain any of the following sequences “N”, “AAAA”, “TTTT”, “GGGG”, and “CCCC”. For each candidate, the cutting position relative to the length of common coding sequence was used to calculate percentage peptide.

**Step 3: Estimate on-target cutting efficiency**

For every candidate along with its percentage peptide, the on-target efficiency was estimated on the Eddie supercomputing cluster (UoE computing facility) using Azimuth 2.0 developed by Microsoft and the Broad Institute^1^. For each gene, all guides were then ranked based on their cutting efficiency from high to low.

**Step 4: Extract off-target sites and estimating off-targeting efficiency**

On the Eddie cluster, the fasta sequences for UMD3.1.1 and the Y chromosome from btau5.01 were indexed by the bwa 0.7.12-r1039 “index” sub-command. All CRISPR candidates were then aligned back to the indexed genome using the “aln” sub-command to identify and extract 20bp sequences with up to three mismatches to the candidate. Only sequences immediately upstream of “NGG”, “NAG” and “NCG” were regarded as potential off-targets and the cutting efficiency at every off-target relative to the on-target was estimated by the CFD package, also developed by Microsoft and the Broad Institute^1^.

**Step 5**: **Select guides**

For each gene, between 4 to 5 guides were selected for inclusion in the final library by processing the ranked list from Step 3 following these rules: **1**. they cut closest to the 5’; **2.** They had the highest on-target efficiency; and **3.** they had the least off-targets with CFD > 0.2 that reside in exons. For some genes, multiple rounds of selection with gradually relaxed rules were needed to identify enough guides; the final list of targeting guides contained 94,000 guides in total.

**Step 6: Generate non-cutting control guides**

A custom python script was used to generate one million random 20bp sequences but only those that met all criteria specified in Step 2 were selected as potential control guides. Sequences selected were also aligned back to the genome as described in Step 4 and only those that align to one or two non-exon sequences with CFD scores less than 0.05 were included in the final 2,000 control-guide list.

**Step 7**: **Synthesize the library as single strand oligos**

The targeting and non-targeting guide sequences were combined, and adapter sequences added to the 5’ and 3’ ends as follows: 5’- GCAGATGGCTCTTTGTCCTAGACATCGAAGACAACACCG-N_20_-GTTTTAGTCTTCTCGTCGC -3’. The list of 96,000 oligos was sent to Twist Bioscience (San Francisco, California) for synthesis and the oligos were delivered as a desalted pool in a single tube.

# Library cloning

**Genome-wide library cloning:** The pool of 80-mer oligos was PCR amplified to produce full length dsDNA segments using primer set 79-mer-L1+79-mer-U1 for cloning the g2 library and set 79-mer-L1v2+79-mer-U1 for the g5 library (see Table D of this document for primer sequences). Purified CRISPR containing PCR products were then ligated into a linearized vector, pKLV-U6gRNA (BbsI)-PGKpuro2ABFP (a gift from Kosuke Yusa)(1) for the K2g2 library or pKLV2- U6gRNA5(BbsI)-PGKpuro2ABFP-W^2^ for the K2g5 library (Addgene ID:67974), using BbsI-HF and T4 ligase. The PBg2 and PBg5 libraries were cloned using the same protocol. These vectors contain a hU6 promoter to drive CRISPR expression, a selection marker Puro_2A_BFP for titration and enrichment of transduced cells, and lentiviral elements for viral packaging or PiggyBac repeats for transposition. Ligated products from 12 parallel reactions were pooled, cleaned and electroporated into 4x25ul of electro-competent cells After one hour of recovery at 37° Celsius, 5ul of bacteria were used for plating on Agar plates with 100ug/ml Ampicillin with serial dilutions to estimate coverage. The bulk remaining culture was maintained in 500ml 2XLB with 100ug/ml Ampicillin and plasmid maxipreps were prepared from the overnight culture using the Qiagen Maxi plus kit. The depth, coverage and accuracy of the libraries was determined using the Illumina NextSeq 500 with the SR75 high output kit.

# Focused CRISPR library design and cloning

Candidate genes were chosen by filtering genes that satisfy the following criteria: p-value < 0.05 and |l2fc| >=0.75 based on results from Neg v.s. High and Low v.s. High comparisons of the 2^nd^ genome wide screen. 83 genes with |l2fc| <=0.06 and ranked between 10,000 and 13,000 were chosen as neutral genes. The final library contained 6,071 guides in total with eight guides targeting each of the 679 candidate genes and 83 neutral genes. It also included 326 non-targeting control guides. The oligo pool was ordered from Twistbio and they were cloned into the lentiviral library using the g5 scaffold as with the genome wide library. The plasmid library was packaged into lentivirus and transduced into the Cas9+/+ cells before BoHV-1 challenge.

# CRISPRi guide RNA design and cloning

The CRISPRi/3i guides were designed using a similar pipeline to that used for the btCRISPRko.v1, utilizing the 400bp genomic sequence immediately downstream of the TSS rather than protein coding sequence. The TSS information were extracted and combined from both the Ensembl (release-95) and NCBI (GCF_002263795.1) annotations of the Bos taurus assembly ARS-UCD1.2. For genes with two TSS annotations within 1 kb of each other, the downstream TSS was chosen. For genes with the two predicted TSS annotations greater that 1 kb apart, the NCBI annotation was used for the design.

The PiggyBac vector used to deliver CRISPRi, PB-U6g5_PGK_Puro2aBFP, was constructed by cutting out the segment containing the hU6 sgRNA expression cassette and the Puro2aBFP selection marker from pKLV2-U6gRNA5(BbsI)-PGKpuro2ABFP-W (gift from Dr Kosuke Yusa, Addgene # 67974) and cloning it into a PiggyBac backbone. The transposase expression vector pCMV-hypBase was shared by Dr. Kosuke Yusa while he was based at the Wellcome Sanger Institute. The H1 and mU6 donor plasmids were created by inserting PCR fragments with scaffold and H1/mU6 sequences into the Zero Blunt Topo vector.

To prepare the fragments, the dsODNs containing the guides were annealed by mixing two complementary oligonucleotides and cooling from 95°C to 25°C at 0.1°C/second; the vector backbone and H1 mU6 donor plasmid were digested with BbsI and fragments of the predicted sizes were gel extracted. The six fragments (**Fig 4**) were ligated using T4 ligase then transformed into Stabl3 competent cells. Colonies were pre-screened with bacterial PCR and Sanger sequencing verified before plasmid purification.

# Tissue culture

MDBK cells were maintained in DMEM culture media supplemented with 2% or 5% horse serum, 0.5% Pen/Strep, 1% L-Glutamine, 1% Sodium Pyruvate, and 1% NEAA, in a 37°°C incubator with 5% CO_2_. When confluent, cells were passaged 1:6 or 1:9 using Trypsin and became confluent in 2-3 days. Cells were frozen at concentrations between 1-5x 10^6^ /ml in freezing media made of 10% TC grade DMSO and 90% horse serum or culture media and transferred to -150° Celsius after overnight storage at -80° Celsius. HEK293FT cells are maintained in DMEM supplemented with 10% fetal calf serum and 0.5% Pen/Strep, in a 37°°C incubator with 5% CO_2_. When confluent, cells were trypsinized for 1 minute and expanded 1:9 for subsequent lentivirus packaging.

# Transfection

0.5-2 x10^6^ MDBK cells were transfected with plasmid DNA, TALEN mRNA, or sgRNA using a Neon electroporator with the following program: 1200v, 30ms, 2 pulses.

For Cas9 targeting to rosa26, 1ug of TALEN mRNA pair #1.6 and 5ug of HDR template were co-transfected into wt MDBKs. After 2 days of incubation at 33°°C, cells were recovered for one day at 37°C before dilutional cloning and colony isolation.

For generating Cas9+/+;TRIM5a-/- clones, cells transfected for Cas9 KI from above were sequentially transfected with 1ug of TRIM5α TAL1L+1R mRNA and clones were isolated and genotyped for both Cas9 KI and TRIM5α KO.

For generating knockout clones using CRISPR sgRNA, 1ug *in vitro* transcribed sgRNA was transfected into Cas9 +/+ MDBKs and cells recovered at 37°°C for two to three days prior to dilutional cloning. For knockouts using PiggyBac transposons, 2.5ug of PiggyBac plasmids carrying hU6 promoter driven gene specific sgRNAs was mixed with 1ug of pCMV-hypBase transposase for co-transfection into Cas9 +/+ MDBKs.

To create rescued cell lines, VPS51, VPS52, and VPS53 KO clones are co-electroporated with a PiggyBac vector carrying the cDNA expression cassettes and pCMV-hypBase supplying PBase at the ratio of 3ug: 1ug per transfection.

# Serum free lentivirus library packaging, titration and transduction

Once passing quality control, the lentivirus library was packaged in HEK293FT cells using the Calcium Phosphate transfection method (Fig BA and BB in S2 Text). One day prior to transfection, 20x 15cm petri dishes were each seeded with 5 x10^6^ early passage HEK293FT cells. 2 hours before transfection on the second day, cells were fed with 10 ml prewarmed fresh media for each plate. Cells were then transfected, using a well-established Calcium Phosphate protocol(2), with the CRISPR library plasmid pool and the two packaging plasmids pMD.2 and psPAX2. The day after transfection, cells were fed with 20ml serum free media buffered with HEPES. 24 hours after media change, the supernatant with cell debris from each plate was combined, cleared by low-speed centrifugation at 300xg for 5 minutes and filtered through a 0.45um low protein binding membrane. The lentivirus stock was then aliquoted into 50ml conical tubes and stored at -80° Celsius until the day of library transduction.

For titration, 3x10^5^ MDBK cells were plated on one 6-well plate 10 hours prior to transduction. 100ul, 80ul, 40ul, 20ul, 10ul or 0ul lentivirus supernatant were mixed separately with 0.8ug/ml Polybrene in culture media with 1ml in total volume in 1.5ml Eppendorf tubes. The culture media was then aspirated from the 6-well plate and the lentivirus dilutions added to the cells immediately. 24 hours after incubation, the virus was then removed and 2ml of fresh media added to each well. The cells were cultured for another day prior to FACS to detect percentage of cells with BFP. The quantity of virus used for the well with ≤30% transduced cells was chosen to calculate the amount of virus needed for the screen at a MOI=0.3.

To produce library transduced cells for the screen, the lentivirus supernatant was used for transduction without prior concentration. 10 hours prior to transduction, a total of approximately 9x 10^7^ TRIM5 -/-; Cas9+/+ MDBKs were plated in 12X T175 flasks. Cells were then transduced at MOI of ~ 0.3 by diluting viral stocks into 10ml total volume using culture media containing 2% horse serum and 0.8ug/ml Polybrene (final concentration). 24 hours after transduction, the virus was replaced by fresh media with 2% horse serum. 48 hours after transduction, the media was replaced with media containing 1.8ug/ml Puromycin for 8-10 days to obtain a pure population of transduced cells. The transduction was repeated three times and cells from every passage were frozen in culture medium supplemented with 10% DMSO and stored at -150° Celsius. To ensure that the libraries are representative and that all the sgRNAs are represented, genomic DNA is isolated from passage 4 transduced cell pools and using primers directed against the lentiviral construct flanking the sgRNA sequences (Fig N in S2 Text), the inserted sequences are amplified and sequenced, providing counts of sgRNA occurrences.

# TALEN assembly and *in vitro* transcription of TALEN mRNAs

TALENs were assembled the using Golden Gate TALEN and TAL Effector Kit 1.0 following the published protocol(3). In place of the destination vectors included in the kit, PMC-DeltaTAL was used in the second reaction. This plasmid was constructed by transferring the DeltaTAL fragment in between restriction sites(4) into the multiple cloning site of pMC128(5). TALENs were sequence confirmed following bacterial PCR. To transcribe the TALEN DNA pairs into mRNA, 10ug of the left TALEN was combined with 10ug of the right TALEN and linearized overnight with 20U of NotI-HF. The digest was treated with RNAsecure and cleaned up using the MinElute PCR cleanup kit. 1ug eluted linear DNA was then used for *in vitro* transcription and polyA tailing with the HiScribe T7 ARCA mRNA Kit. The polyA mRNA products were purified using a RNeasy column, nanodrop tested for concentration, aliquoted and stored in -80°C.

# Individual sgRNA cloning and *in vitro* transcription of sgRNAs

Individual guides were cloned into either PB_U6gRNA5-PGKpuro2ABFP-W or pKLV2-U6gRNA5(BbsI)-PGKpuro2ABFP-W by BbsI digestion and T4 ligase ligation as published(6).

To generate knockout clones by transfecting sgRNA, sgRNA was synthesized by *in vitro* transcription using the HiScribe T7 Quick High Yield RNA synthesis kit. Briefly, to prepare the DNA template for transcription, a PCR was carried out using ttaatacgactcactatagGN19GTTTAAGAGCTATGCTGGAAAC as forward primer, AAAAGCACCGACTCGGTGCC as universal reverse primer and pKLV2-U6gRNA5(BbsI)-PGKpuro2ABFP-W serving as PCR template. The forward primer contains a T7 promoter (lower case), G plus last 19 bp of desired CRISPR sequence (GN19), and partial sequence of the g5 scaffold (underscored sequence). The PCR product was purified using the MinElute PCR cleanup kit and the T7 promoter in the ~110bp amplicon used to transcribe the downstream sgRNA. The *in vitro* transcription was done according to the kit manual with 100ng PCR product as template and overnight incubation. The reaction was purified with a RNeasy column, and the sgRNA tested for concentration using a nanodrop spectrometer(7), aliquoted and stored at -80°C.

# Gene editing efficiency testing by T7E1 digestion and TIDE analysis

Two to three days after transfection with TALEN mRNA or individual CRISPR sgRNAs, crude genomic DNA was extracted from cells using QuickExtract DNA Extraction Solution following the product manual. The extract was diluted 1:10 in nuclease free water and 1ul of the dilution used for PCR using primers flanking the target site and Phusion HF polymerase with supplied buffer, producing amplicons between 300-800bp in size. 2ul of PCR reaction is ran on 2% Agarose gel to estimate amplicon concentration. Without purification the calculated volume of PCR reaction containing ~200ng PCR product based on the agarose gel was denatured at 95°°C for 5 minutes and re-annealed by dropping the temperature to 25°C at 0.1°C per second in a thermal cycler, at the end of the program the temperature was reduced to 4°C. Right after re-annealing, 1ul T7 Endonuclease I was added to the PCR reaction directly and incubated at 37°C for 30 minutes. Immediately after incubation, the reaction was analyzed on a 2% Agarose gel. The gel image was processed by the Densitometry function of ImageJ and the percentage editing was calculated as described previously(4). The editing efficiency was also examined by TIDE analysis (<https://tide.deskgen.com/> )(8) following Sanger sequencing of purified PCR products.

# Generating the Cas9+/+, and Cas9+/+; TRIM5 -/- clones

To generate Cas9+/+ clones, TALEN pair TAL1.6 was chosen out of all TALENs and CRISPRs designed that target first intron of the rosa26 locus. It cuts with 52% cutting efficiency based on T7E1 assay and 1ug of the mRNA was co-transfected with 5ug plasmid pMT2.0_bovRosa26_EF1a_Cas9_blast using Neon. After 2 days of incubation at 33°C, cells were recovered at 37°C for two days and then selected with 10mg/ml Blasticidine for four days. Single cell clones were isolated by dilution cloning and genotyped by PCR, homozygotes were expanded and stored in -150°C.

To generate Cas9+/+;TRIM5-/- clones, the population of cells after Blasticidine selection from above was transfected with 1ug of TAL1L+1R mRNA, then cultured and recovered as above. New clones were isolated by dilution cloning and they were screened using a GFP expressing lentivirus. Only those that appeared to have higher % of GFP+ cells judged by eyes were genotyped for both Cas9 KI and TRIM5a KO by PCR and sequencing. Clones homozygous for both Cas9 KI and TRIM5a KO (Cas9+/+;TRIM5-/-) were screened with GFP lentivirus again and the transduction efficiency was measured by FACS to confirm phenotype. Only those with the best morphology and confirmed with enhanced transduction efficiency were expanded for the screen.

# Colony isolation by dilutional cloning and genotyping

For plasmid-based gene targeting, cells are selected for four days with 10mg/ml Blasticidin or 1.8ug/ml Puromycin depending on the construct. After drug selection, cells are plated at 50-100 cells per dish on 10cm dishes for colony formation. After ~7 days of incubation, colonies are picked, expanded and genotyped by Sanger sequencing and TIDE analysis. For TALEN mRNA or sgRNA based editing, after 2-3 days of recovery from transfection, cells are plated on 10cm dishes at the same density for colony formation without any drug selection.

# Production of rescue cells

cDNA sequences for bovine/human VPS50-54 subunits were amplified from total cDNA reverse transcribed from wt MDBK cells and cloned into a PiggyBac vector after a CAG promoter followed by a Puromycin selection cassette connected via an IRES sequence. Representative VPS50-54 KO clones were then transfected with the cDNA expression vectors and pCMV-hypBase using Neon. Cells were then selected with Puromycin 48 hours after transfection and expanded for experiments.

# BoHV-1 infection

For the screening, 10^6^ cells were plated in each T175 flask (9 flasks per repeat) 12 hours prior to infection (passage 4 or 5 with 9-10 days of puro selection and culture post transduction). Cells in each flask were infected with GFP tagged BoHV-1 at a MOI=2 in media containing 2% horse serum. After one hour the virus inoculum was removed and replaced with 20ml of fresh media with 2% Horse Serum. This time point was counted as 0-hour post infection (0hpi). BoHV-1 infections for single gene studies are scaled down with the quantities of viruses calculated based on the desired MOI.

# Plaque assays

One day before plaquing, approximately 5 x10^5^ cells from each cell line are plated in 2ml culture media with 2% horse serum per well on 6-well plates. One day later, cells were infected with 10-fold serial dilutions of wt or GFP tagged BoHV-1 virus in 1ml of media with 2% horse serum. After one hour the virus inoculum was removed and 2mls of Avicel overlay (culture media with 2% horse serum and 0.5% Avicel) was added to each well. The plates are returned to the incubator, and four days later, cells were fixed by adding 1ml 10% Neutral Buffered Formalin. After one hour of incubation at room temperature, the liquid mix is removed and 1ml 0.1% Toluidine Blue is added to each well. After staining for one hour at room temperature, the wells are washed gently with tap water and inverted to dry overnight. Plaque numbers are counted under an inverted microscope and the plates are also scanned using an Epson document scanner. The scanned images were processed by ImageJ to count and measure sizes of plaques. Plaque assays for AIHV-1 and HSV-1 were conducted using similar protocols.

# FACS sort

To prepare cells for sort, media was removed carefully at the appropriate time point without dislodging cells and 1.5ml 0.5% Trypsin was added to the flasks immediately without PBS wash. After 5 minutes of incubation at 37° Celsius, trypsin was neutralized with 8ml of culture media and the cell suspension harvested. The flasks were rinsed with 10mls of PBS and trypsinized again for 3 minutes to recover the residual cells. All cell suspensions from the two rounds of trypsinization and the PBS washes were combined, centrifuged at 1200g for 5 minutes, washed once with 50ml of PBS and recentrifuged. The cell pellet was then resuspended in 8mls of PBS containing 2mM EDTA. The cell suspension was kept on ice and filtered with a 0.22um nylon mesh prior to sort and maintained at 6°C during sort. Four fractions of live cells, GFP negative, ~10% GFP low, ~10% GFP Medium, and ~10% GFP High cells are collected. Collection tubes are pre-filled with 200ul horse serum prior to sort.

# genomic DNA isolation from MDBK cells and PCR for NextSeq

After FACS sort, collected cells were centrifuged at 1200xg for 5 minutes, washed once with PBS and the pellet stored at -80° Celsius until genomic DNA isolation. Based on the number of cells recovered, genomic DNA was isolated from the samples using one of the following kits: Quick-DNA Microprep kit, Quick-DNA Miniprep Plus Kit, DNeasy Blood & Tissue kit, NucleoSpin Blood L Column, or NucleoSpin Blood XL Column. For non-infected control samples, fragment enrichment by HindIII-HF digest and gel extraction was conducted to reduce the number of PCR retractions required to prepare samples for Illumina sequencing. Briefly, 300ug of genomic DNA was digested with 2,000 units of HindIII-HF overnight for 20 hours and resolved on a 0.7% Agarose gel. The fragment containing the hU6-sgRNA cassette is 1485bps, a gel slice containing DNA in the range of 1.2kb -1.8kb was excised from the gel and the DNA extracted from the slice using the NucleoSpin Gel and PCR Clean-up Kit.

To prepare samples for NextSeq, 1- 2ug genomic DNA or 0.5-1ug enriched genomic DNA fragments were used as template in the 1^st^ PCR per reaction, with limited cycles for faithful representation of the original gRNA distributions in the samples. The PCR was performed using Q5 Hot Start polymerase and a cocktail of ten forward primers containing the staggering sequences (primers PCR_F1 to PCR1_F10, Table E of this document) and the reverse primer PCR1_R (Table E). For every sample, 5ul from each PCR reaction was pooled and cleaned up using a PCR cleanup column. The cleaned-up product was then used in a 2^nd^ PCR with 10 cycles for barcoding, using NEBNext Ultra II Q5 polymerase and a unique combination of one forward primer idx_Sxxx (Table E) and one reverse primer idx_Nxxx (Table E). The 2^nd^ PCR reaction was then cleaned up using 1 volume AMPure XP beads on a magnetic stand and eluted with EB buffer from a Qiagen miniprep kit. The purified 2nd PCR product is quantified using a Qubit dsDNA HS Assay Kit and a Qubit 2.0 machine. The products were also visualized, and quality confirmed by running 2ul of each purified product on a 2% Agarose gel. Purified products for all samples were pooled into and sent for sequencing on a NextSeq 500 machine. The sample was sequenced using a Single Read 75bp High Output Kit v2 with 25% PhiX spike in, resulting in ~320 million reads passing quality control. The reads are then demultiplexed and assigned to each sample based on their unique barcode combinations.

All illumine sequencing was carried out on a Nextseq 500 machine at the Edinburgh Clinical Research Facility (ECRF) using NextSeq 500/550 High Output Kit v2.5 (75 Cycles). A spike-in of 25% PhiX was added to the sample mix to increase sequence diversity.

# NextSeq reads processing and data analysis

For each sample, the sequencing reads were checked for quality using FastQC v0.11.4 and trimmed using cutadapt v1.16 from the 5’ until sequence CGAAACACCG (inclusive) and from the 3’ until GTTTAAGAGC (inclusive), discarding all untrimmed sequences and keeping only trimmed reads with 20bp in length. The copy number of trimmed reads matching each guide in the CRISPR library was then counted using the *count* sub-command from the MAGeCK package v 0.5.8(9). Pairwise comparisons of guide copy numbers between samples were done using the *test* sub-command from MAGeCK. Candidate genes with significantly depleted or enriched guides were selected based on a cut-off of adjusted P-Value < 0.005, FDR<0.1 and l2fc>=1 or <=-1.

# Gene Ontology analysis

All GO enrichment analyses were completed using PANTHER Overrepresentation Test (Released 20190711) and GO Ontology database (Released on 2019-07-03) hosted on website <http://geneontology.org/>. The queries were conducted using FISHER as the Test Type and all genes in database for Homo sapiens as Reference List. We chose the Homo sapiens list instead of that for Bos Taurus due to better data availability.

# STRING clustering analysis

STRING analysis of lists of candidate genes were queried using the “Multiple Proteins by Names/Identifiers” search box against the database for Bos Taurus on: <https://string-db.org/cgi/network.pl?taskId=4414a1leHqHx>.

Default values were used for all settings except the following: meaning of network edges = molecular action; Clustering method = MLC clustering.

# Viral genomic DNA sample collection, quantification and genome to pfu growth curves

50K/well VPS52KO cells and control cells (Clone P) were plated on 24-well plates and infected with the GFP tagged BoHV-1 at MOI=0.1 or MOI=3 the second day. Following infection, total virus, virus from tissue culture supernatants or cell pellets were harvested at specified time points post infection. 5ul of each sample was lysed with an equal volume of QuickExtract DNA buffer and treated according to the manufacturer’s instructions. Viral DNA copies were then determined by qPCR with primers set UL23_qPCR_F + UL23_qPCR_R using [LightCycler 480 SYBR Green I Master](https://shop.roche.com/shop/store/ProductDisplay?catalogId=10001&partNumber=3.5.8.1.1.2)reagents and samples were run in a LightCycler 480 machine. For each repeat, the highest Ct value was used as base level (copy number set at 1 for the corresponding sample) and the relative genome copy number for each sample was calculated as 2^-(sample Ct – highest Ct). The titer of each sample was also obtained by plaquing the virus samples on wt MDBK cells. For each sample, a ratio between the genome copy number and virus titer was calculated as the genome to pfu ratio. And for each repeat, the lowest genome to pfu ratio was used as a normalization factor and all genome copy number to virus ratios were divided by this factor to obtain normalized genome to pfu ratio prior to plotting.

# Reverse transcription, Quantitative PCR of viral transcripts and viral mRNA/viral DNA growth curves

To compare the viral mRNA levels by reverse transcription and qPCR, total RNA from cells was isolated using Trizol LS (Qiagen, Hilden, Germany). Then RNA was DNase-treated using Turbo DNA-free kit (Invitrogen), and cDNA was synthesized using a QuantiTect reverse transcription kit (Qiagen, Hilden, Germany). Quantitative real-time qPCR of cDNA was carried out using the LightCycler 480 System and LC480 SYBR Green 1 Master (Roche) following the protocol provided by the manufacturer. Relative BoHV-1 ICP0, Circ, UL23 and gB mRNA/cDNA levels were quantified by qPCR using 18S rRNA internal controls to normalize template input and presented as fold change against the CTRL cells. Gene differential expression between samples was calculated using 2-ΔΔCt method. The viral genomic DNA copies were also obtained using the QE DNA extraction buffer as above. A ratio between the copy number of mRNA/cDNA to the viral DNA was also calculated and normalized to the lowest ratio within each repeat prior to data plotting.

# VP26-GFP growth tracing

30,000 cells were seeded in RPMI media without Pheno red plus 5% HS on a 96-well clear bottom dark walled culture plate 12 hours prior to reading. For each cell line, four replicates were set up. The outside wells were avoided to reduce bias from evaporation. Cells were infected by the GFP tagged BoHV-1 virus either at MOI=3 or MOI=0.1 and the first reading was taken at 0 h.p.i. The cells were incubated in 5% CO2 at 37 °C while the Fluorescent Intensity (FI) from each well was recorded by CLARIOstar Plus Microplate Reader (BMG LabTech) every 10 minutes for 72 hours.

# Cloning and transfection of constructs expressing candidate proteins of BoHV-1 to identify the FLI antibody target

**Cloning of constructs:** ORFs of BoHV-1 candidate proteins identified by Mass Spec (i.e. gB, gC, gD, gE, gG, gH and VP8) were PCR amplified using the GXL PrimeSTAR polymerase with 9% DMSO spike in, from genomic DNA of BoHV-1 with primers based on the genomic sequence of Strain cooper (NCBI genome ID KU198480.1). For each ORF, the PCR primers were designed to flank the ORF with a FLAG tag immediately upstream of the stop codon and overlapping sequences for cloning by Gibson Assembly (Table D of this document). Using Gibson assembly, the ORFs were then cloned into a PiggyBac vector based on PB-SAM (Addgene ID: 102559, PCR amplified by primers listed in Table D, flagPB5_F and startPB3_R), replacing the dCas9-blast ORF with the ORFs of BoHV-1.

**Transfection:** 2ug of each plasmid construct cloned above (PB-gBflag to PB-VP8flag) was transfected into HEK293FT cells plated on a 24-well plate at 50% confluency, with 5ul Lipofectamine 2000 according to the manufacture instruction.

**Sample harvesting and western blotting:** 24 hours post transfection, cells were lysed with 2x Laemmli buffer with DTT and boiled at 95 degrees Celsius for 5 minutes prior to being resolved on a 10% SDS PAGE gel. The proteins were then transferred onto a nitrocellulose membrane with 0.45um pore size and blocked with 5%BSA for 1 hour in room temperature. They were then stained with the primary FLI antibody (1:100 dilution, raised in mouse) and the anti-flag antibody (Sigma, raise in rabbit) followed by Licor Donkey anti-mouse Dylight 680 (for the FLI primary antibody) and Donkey anti-rabbit Dylight 800 secondary antibodies prior to imaging.

# Concentration of viruses and western blot staining for VP8 to VP26-GFP ratios

**Infection:** WT, Cas9+/+ non-KO cells (Clone P) or VPS52 knockout cells were plated on T75 flasks at 50% confluency. One day later, cells were infected with GFP tagged BoHV-1 at MOI=3. 4hpi, the inoculum was taken off from each flask and the cell monolayer was washed with 10ml pre-warmed PBS three times and 6ml of media (DMEM + 5% horse serum + 1% pen/strep + 1% L-Glutamine) was added after washing. Flasks were incubated for a further 48 hours at 37°C with 5% CO2. At the time of harvest, media was pipetted up and down to flush the flask and the cell/media suspension was then transferred into a 15ml conical tube and centrifugated at 1200rpm for 5min.

**Sample harvest:** 3ml of each supernatant sample was concentrated in a Beckman benchtop ultracentrifuge using a TLA100.3 rotor with 30% sucrose in PBS spun at 125,000g or 48,000 rpm for 1.5hrs. After spinning, the supernatant was taken off using a p1000 pipette and the virus pellet was resuspended in 30ul of 2X Laemmli buffer containing 0.2M DTT. The cell pellets from each infection were also lysed with 500ul of 2x Laemmli containing DTT. All lysates were then boiled at 95 °C for 5 minutes before running on a PAGE gel.

**Western Blot staining and densitometry analysis:** Samples were resolved on a 10% SDS PAGE gel ran at 110V for 1.5hours. Proteins were then transferred onto nitrocellulose membranes (0.45um in pore size) using the bio-rad semi-dry Turbo transfer system and blocked for at least 1 hour in PBST (1xPBS with 0.1% Tween-20) containing 5% Bovine Serum Albumin (BSA). The membrane was sequentially incubated with primary antibody against VP8 (1:2000 dilution of tissue culture supernatant of hybridomas secreting antibody against envelope protein of BoHV-1 strain "Schönböken", kindly provided by the FLI institute in Germany) and then against GFP (JL-8, Takara Bio) in 1x PBST containing 2.5% BSA. For the cell lysates, the membrane was also blotted with rabbit anti beta-actin antibody. The membrane was then hybridized with secondary antibody Licor IRDye 680RD Donkey anti-Mouse IgG (for detection of VP8 and GFP) and/or Licor Donkey anti rabbit Dylight 800 secondary antibody (for the detection of beta actin in the cell lysates) prior being imaged on the Licor Odyssey Fc Imaging System. The images were then converted into black and white, and the intensity of each target band was quantified using the Fiji software. The ratio of VP8 to GFP or GFP to beta actin was calculated by dividing the densitometry reading of VP8 by that of GFP or GFP by that of beta actin before being normalized to the ratio obtained for WT cells (which is set as 1).

# Coomassie staining and Mass spec sample preparation

**Coomassie staining:** Protein lysate samples were resolved on 10% SDS PAGE gels for 1.5 hour at 100V. Afterwards, the gel was fixed and stained with buffer containing 50% Methanol, 10% Acetic Acid and 0.1% Coomassie Blue R250 in water overnight. The gel was then de-stained in 10% acetic acid, 50% methanol and 40% water till the background was low.

**Gel excision for Mass Spec**: After de-staining, for each sample/ lane, a gel piece containing proteins with masses between 90kD and 110kD were excised using a surgical blade and deposited into an Eppendorf tube prior to protein elution and Mass Spec.

# Immunofluorescent staining and confocal imaging

One day before infection, 50,000 WT, Cas9+ or 52KO cells were plated on each glass cover slip on 24-well plates. One day after plating, cells were infected with the GFP tagged BoHV-1 virus at MOI=1. At 0,3,6,10 hpi, coverslips were washed 2x with PBS and fixed with PBS containing 2% Formaldehyde for 10 minutes. The coverslips were then rinsed twice with PBS containing 1% FBS, permeabilized by 100% ice cold Methanol for 10 minutes and then stored at 4 °C in 1% FBS containing PBS overnight prior to antibody staining. For staining, coverslips were first incubated with 1:2000 anti-VP8 antibody (raised in mouse hybridoma, FLI Institute) and 1:2000 anti-GM130 antibody (raised in rabbit, ab52649, abcam) for one hour in room temperature. After 3x washing with PBS, the coverslips were stained with Donkey anti-mouse secondary antibody conjugated with Donkey anti-mouse secondary antibody conjugated with Alexa 594 and Donkey anti-rabbit secondary antibody conjugated with Alexa 647 for 30 minutes. The cover slips were washed 3x with PBS, the first wash lasting for 10 minutes with 1:10,000 DAPI stain spike in. The cover slips were then mounted on glass slides using Pro-long Gold mounting media (Thermo Fisher) and cured at room temperature overnight prior to imaging on a Zeiss LSM710 confocal microscope for the detection of DAPI, VP26-GFP, VP8 and cis-Golgi marker GM130.

# Statistical analysis

Unless otherwise stated, all statistical comparisons among three or more groups of cell lines in single timepoint experiments were conducted using the Graphpad Prism version 9 or “Analysis Tool ANOVA: Single Factor” provided by Microsoft Excel with Version 16.34 (20020900) followed by Dunnett’s multiple comparisons (**Figs 1C, 4H, 6B, 6C, 6E, 6F, 6G, 6H, 8D,88E, 8G, 8H, and 8I**). For time course experiments, all comparisons were conducted using two-way ANOVA (mixed model) followed by a student’s t-test for each time point (**Figs 7 and S in S2 Text**). p-values are denoted as ****: p<0.0001; ***:0.0001 to 0.001; **:0.001 to 0.01; *: 0.01 to 0.05; n.s.: not significant with p>=0.05.

#

# Table A. Plasmids used in the study

| **Plasmid name** | **Source** | **Purpose** |
| --- | --- | --- |
| pKLV-U6gRNA(BbsI)-PGKpuro2ABFP | Gift from Kosuke Yusa | Lentivirus g2 Library cloning |
| pCMV-hypBase | Gift from Kosuke Yusa | Piggyback transposase |
| pKLV2-U6gRNA5(BbsI)-PGKpuro2ABFP-W | Addgene # 67974 | Lentivirus g5 library cloning |
| lentiGuide-Puro | Addgene # 52963 | Lenti delivery of sgRNA |
| pSpCas9(BB)-2A-GFP (PX458) | Addgene # 48138 | CRISPR/Cas9 expression |
| PMD2.G | Addgene # 12259 | lentivirus packaging |
| psPAX2 | Addgene # 12260 | lentivirus packaging |
| pHIV-EGFP | Addgene # 21373 | Control lentivirus |
| PB-SAM | Addgene # 102559 | cDNA expression backbone |
| pMT2.0_bovRosa26_EF1a_Cas9_blast | this study | Cas9 targeting to rosa26 |
| PB_U6gRNA2-PGKpuro2ABFP-W | this study | PiggyBac g2 Library cloning |
| PB_U6gRNA5-PGKpuro2ABFP-W | this study | PiggyBac g5 library cloning |
| PB_U6gRNA2-CAGpuro | this study | PiggyBac delivery of sgRNA |
| PMC-DeltaTAL | this study | TALEN assembly |
| PMC-bovRosa26_1.6L | this study | Rosa26 targeting |
| PMC-bovRosa26_1.6R | this study | Rosa26 targeting |
| PMC-bovTRIM5a_2.2L | this study | TRIM5a targeting |
| PMC-bovTRIM5a_2.2R | this study | TRIM5a targeting |
| PB_VPS51R | this study | cDNA overexpression |
| PB_VPS52R | this study | cDNA overexpression |
| PB_VPS53R | this study | cDNA overexpression |
| PB_VPS50R | this study | cDNA overexpression |
| PB_VPS54R | this study | cDNA overexpression |
| PB_huVPS51R | this study | cDNA overexpression |
| PB_huVPS50R | this study | cDNA overexpression |
| PB_huVPS54R | this study | cDNA overexpression |
| PB_gBflag | this study | cDNA overexpression |
| PB_gCflag | this study | cDNA overexpression |
| PB_gDflag | this study | cDNA overexpression |
| PB_gEflag | this study | cDNA overexpression |
| PB_gGflag | this study | cDNA overexpression |
| PB_gHflag | this study | cDNA overexpression |
| PB_VP8flag | this study | cDNA overexpression |

**Note:** Plasmids generated in this study are available upon request.

# Table B. Individual CRISPRs and TALENs used in this study

| **Nuclease name** | **Sequence** | **Purpose** |
| --- | --- | --- |
| Rosa26_1.6L | NN NN NN HD HD NG NI NN NI NI NN NI NI NG HD HD HD NG | Rosa26 targeting |
| Rosa26_1.6R | HD NN NI HD NI NG NN NN NI NN NN HD NN NI NG NN NI HD NN NI | Rosa26 targeting |
| TRIM5_1L | NI NN NN NI NN NG NN HD NI NI NI NG NN NG NG NN | TRIM5 KO |
| TRIM5_1R | NG HD NG HD NI HD NG NG NN NG HD NG HD NG NN | TRIM5 KO |
| VPS50_sgRNA | AGGGACCCGGAGACTCTCAA | KO |
| VPS51_sgRNA | GTTGTAGTTCTCATAGACCA | KO |
| VPS52_sgRNA | GGAGCTTGTTGATGGTCTCG | KO |
| VPS53_sgRNA | GTTCAGCGTTGTGATCGAGG | KO |
| VPS54_sgRNA | AGGCTGAGTCTGGTGTACAG | KO |
| CTNNB1 g1 | GAGCGGTAAAGGCAATCCTG | KO |
| CTNNB1 g2 | GTTCCCTGAGACGCTAGATG | KO |
| MAPK8 g1 | GTAGTAGCGAGTCACTACGTA | KO |
| MAPK8 g2 | GTCGCTACTACAGAGCACCTG | KO |
| MAPK9 g3 | GACCCTGAAGATCCTCGACTT | KO |
| MAPK9 g4 | GTGGTGCACGCTGTACGAGCC | KO |
| Oct-1 g1 | GCTGGCGGAATGCTGCTGCA | KO |
| Oct-1 g2 | GCTGTATGGGCTGAGACAAG | KO |
| SGK1 g1 | GTGCAGGTAACCCAAGGCAC | KO |
| SGK2 g1 | GCAGCATAGAATCGAGCCCG | KO |
| SGK3 g1 | GTTGTTCTACCATCTCCAGA | KO |
| huVPS50 | gTTGAAGACCTGTCTGCAATG | KO |
| huVPS51 | GACCCGACTGATCTGAACG | KO |
| huVPS52 | gCAACTTGCTTTGAATAGTGA | KO |
| huVPS54 | gATGTAAAAGAGTCCTTTCGA | KO |

# Table C. Oligos used to clone CRISPR3i vectors for gene knockdown

| **Sequence Name** | **Sequence** |
| --- | --- |
| 3iG1_RNGTT::61102289+:NC_037336.1 | CAC CGT CAC AAC AAG ATC CCG CCG GTT |
| 3iG1_RNGTT::61102289-:NC_037336.1 | CTT AAA CCG GCG GGA TCT TGT TGT GAC |
| 3iG2_RNGTT::61102306+:NC_037336.1 | ATC TGG ACA GTT TAG CCA CCG CGG GTT TAA |
| 3iG2_RNGTT::61102306-:NC_037336.1 | GCT CTT AAA CCC GCG GTG GCT AAA CTG TCC |
| 3iG3_RNGTT::61102207+:NC_037336.1 | TGT TGT GGT TCA GAG CGG ACC GCG |
| 3iG3_RNGTT::61102207-:NC_037336.1 | AAA CCG CGG TCC GCT CTG AAC CAC |
| 3iG1_SLC30A1::71867793+:NC_037343.1 | CAC CGG GCT CGG GAG TCT GCC GCG GTT |
| 3iG1_SLC30A1::71867793-:NC_037343.1 | CTT AAA CCG CGG CAG ACT CCC GAG CCC |
| 3iG2_SLC30A1::71867428+:NC_037343.1 | ATC TGT CCT CGG TCC GGG CCA CAC GTT TAA |
| 3iG2_SLC30A1::71867428-:NC_037343.1 | GCT CTT AAA CGT GTG GCC CGG ACC GAG GAC |
| 3iG3_SLC30A1::71867510+:NC_037343.1 | TGT TGG ACC GGC AGC TCA GCG CTG |
| 3iG3_SLC30A1::71867510-:NC_037343.1 | AAA CCA GCG CTG AGC TGC CGG TCC |
| 3iG1_TMEM41B::43311362+:NC_037342.1 | CAC CGG GAT CCT TAC AAC ACC CAG GTT |
| 3iG1_TMEM41B::43311362-:NC_037342.1 | CTT AAA CCT GGG TGT TGT AAG GAT CCC |
| 3iG2_TMEM41B::43311459+:NC_037342.1 | ATC TGG TCG AAA GAT CGC AAA CGG GTT TAA |
| 3iG2_TMEM41B::43311459-:NC_037342.1 | GCT CTT AAA CCC GTT TGC GAT CTT TCG ACC |
| 3iG3_TMEM41B::43311568+:NC_037342.1 | TGT TGA GAC CTG GGA ACC AGC GCG |
| 3iG3_TMEM41B::43311568-:NC_037342.1 | AAA CCG CGC TGG TTC CCA GGT CTC |
| 3iG1_TP53::27388075+:NC_037346.1 | CAC CGA ACT CGA AGC TAA ACA CTT GTT |
| 3iG1_TP53::27388075-:NC_037346.1 | CTT AAA CAA GTG TTT AGC TTC GAG TTC |
| 3iG2_TP53::27388296+:NC_037346.1 | ATC TGG AGC TTA CCC GAA GCT CAG GTT TAA |
| 3iG2_TP53::27388296-:NC_037346.1 | GCT CTT AAA CCT GAG CTT CGG GTA AGC TCC |
| 3iG3_TP53::27388351+:NC_037346.1 | TGT TGG TCG GAC GCT AGA TGA CCC |
| 3iG3_TP53::27388351-:NC_037346.1 | AAA CGG GTC ATC TAG CGT CCG ACC |
| 3iG1_TTC4::91562612+:NC_037330.1 | CAC CGT GGC AGC GCC AAA CCT TGG GTT |
| 3iG1_TTC4::91562612-:NC_037330.1 | CTT AAA CCC AAG GTT TGG CGC TGC CAC |
| 3iG2_TTC4::91562725+:NC_037330.1 | ATC TGG GCT TCC ACG AGA GCC AGT GTT TAA |
| 3iG2_TTC4::91562725-:NC_037330.1 | GCT CTT AAA CAC TGG CTC TCG TGG AAG CCC |
| 3iG3_TTC4::91562593+:NC_037330.1 | TGT TGG TAG AAA CCA CCT ATC CAC |
| 3iG3_TTC4::91562593-:NC_037330.1 | AAA CGT GGA TAG GTG GTT TCT ACC |
| 3iG1_USO1::90560842+:NC_037333.1 | CAC CGA AGA TGA ATT TCC TCC GCG GTT |
| 3iG1_USO1::90560842-:NC_037333.1 | CTT AAA CCG CGG AGG AAA TTC ATC TTC |
| 3iG2_USO1::90561015+:NC_037333.1 | ATC TGG AGG CGG CAT GTA GAC TCC GTT TAA |
| 3iG2_USO1::90561015-:NC_037333.1 | GCT CTT AAA CGG AGT CTA CAT GCC GCC TCC |
| 3iG3_USO1::90560819+:NC_037333.1 | TGT TGG GTA GAG CCC GCG GCT GAG |
| 3iG3_USO1::90560819-:NC_037333.1 | AAA CCT CAG CCG CGG GCT CTA CCC |
| 3iG1_XPO6::25727407+:NC_037352.1 | CAC CGC CGC CGC TTC TGA ACG CGG GTT |
| 3iG1_XPO6::25727407-:NC_037352.1 | CTT AAA CCC GCG TTC AGA AGC GGC GGC |
| 3iG2_XPO6::25727647+:NC_037352.1 | ATC TGA AGT GGC GAG CGC TCG GCG GTT TAA |
| 3iG2_XPO6::25727647-:NC_037352.1 | GCT CTT AAA CCG CCG AGC GCT CGC CAC TTC |
| 3iG3_XPO6::25727309+:NC_037352.1 | TGT TGC TCG GAG GGC TGA CGA GGA |
| 3iG3_XPO6::25727309-:NC_037352.1 | AAA CTC CTC GTC AGC CCT CCG AGC |
| 3iG1_ZBTB1::76649474+:NC_037337.1 | CAC CGC CGG CCG CAG CAG GAA CCG GTT |
| 3iG1_ZBTB1::76649474-:NC_037337.1 | CTT AAA CCG GTT CCT GCT GCG GCC GGC |
| 3iG2_ZBTB1::76649492+:NC_037337.1 | ATC TGA TAA CTG GCA AAT ACT CAC GTT TAA |
| 3iG2_ZBTB1::76649492-:NC_037337.1 | GCT CTT AAA CGT GAG TAT TTG CCA GTT ATC |
| 3iG3_ZBTB1::76649425+:NC_037337.1 | TGT TGA AAT CGG CAG CGG GAA AGG |
| 3iG3_ZBTB1::76649425-:NC_037337.1 | AAA CCC TTT CCC GCT GCC GAT TTC |
| 3iG1_nc_guide74+ | CAC CGC GCG GCG TCC ATA TCA CGT GTT |
| 3iG1_nc_guide74_ | CTT AAA CAC GTG ATA TGG ACG CCG CGC |
| 3iG2_nc_guide95+ | ATC TGC GTA CTC GTA TAC TAC TCG GTT TAA |
| 3iG2_nc_guide95- | GCT CTT AAA CCG AGT AGT ATA CGA GTA CGC |
| 3iG3_nc_guide606+ | TGT TGT GAC GCG TGG TCG AAC CGC |
| 3iG3_nc_guide606- | AAA CGC GGT TCG ACC ACG CGT CAC |

Note: CRISPR sequences are underscored.

# Table D. Primers used for cloning, gene targeting and qPCR

| **Primer name** | **Sequence** | **Purpose** |
| --- | --- | --- |
| 79-mer-U1 | GCAGATGGCTCTTTGTCCTA | g2, g5 library cloning |
| 79-mer-L1 | GCGACGAGAAGACTGTAAAAC | g2 library cloning |
| 79-mer-L1v2 | GCGACGAGAAGACTAAAAC | g5 library cloning |
| btRosa26_NJ_F1 | CGCTGCCTGAAGGACAAGAC | T7/TIDE |
| btRosa26_NJ_R1 | CGGTGTAGCAACGGTCTCAAA | T7/TIDE |
| btRosa26_4k_F1 | TCGTGAGGGTAGGTCTCTCTT | Rosa26 targeting |
| btRosa26_4k_R1 | ACCTGCCAACCAACCAACC | Rosa26 targeting |
| TRIM5a NJ F7 | CCACCCTATTCTCATCATCT | T7/TIDE |
| TRIM5a NJ R7 | GTTGATATACTGAAAAAACATTTG | T7/TIDE |
| VPS50_NJ_F | ATGGTCTCGGTGAAAGAAGGGGAA | T7/TIDE |
| VPS50_NJ_R | CAAGCCCCAAGCCTATTGCATTATA | T7/TIDE |
| VPS51_NJ_F | CCTGGTAACTCTTGAGCTATGCCAC | T7/TIDE |
| VPS51_NJ_R | ATTTCTTTTTAAAATTTCAATGTCT | T7/TIDE |
| VPS52_NJ_F | GTTTTTGTTGTTGCTGCCGTGACTA | T7/TIDE |
| VPS52_NJ_R | TCACGCCTGCCCATCTACTTTCTAT | T7/TIDE |
| VPS53_NJ_F | CGGGTAAAGTCTGCTGAAGAAATGC | T7/TIDE |
| VPS53_NJ_R | TGCCGTTTCCTGCGAGTCATTCAT | T7/TIDE |
| VPS54_NJ_F | TGCTCACCAGATCTCTTTACGTTCA | T7/TIDE |
| VPS54_NJ_R | ACGACTGAGCAACTAAACGGAACTG | T7/TIDE |
| CTNNB1_NJ_F2 | TCCTGGTAGTAATATTGATGCTGT | T7/TIDE |
| CTNNB1_NJ_R2 | TGTCTCTACTTACCTGGTCCTC | T7/TIDE |
| MAPK8_NJ_F2 | TGCCATCAAGCTAATTTCTCAGAT | T7/TIDE |
| MAPK8_NJ_R2 | GCGGGCCCACTATACACTTC | T7/TIDE |
| MAPK9_NJ_F2 | ACCGGAATGTTGGGAACTGA | T7/TIDE |
| MAPK9_NJ_R2 | CTCATCTGTGAACCATGATAATCTA | T7/TIDE |
| Oct-1_NJ_F1 | TGCCATATGAAGTTGGGTAGC | T7/TIDE |
| Oct-1_NJ_R1 | CCTCTGCTCCAGAAATACGC | T7/TIDE |
| SGK1_NJ_F2 | CAGACGGCCGACAAACTGTA | T7/TIDE |
| SGK1_NJ_R2 | AAGCTTGGCCTATGGTCTCC | T7/TIDE |
| SGK2_NJ_F1 | CTGCCTGTAAGTCCTCCGAC | T7/TIDE |
| SGK2_NJ_R1 | TGGGGATCTCACGATGGAGT | T7/TIDE |
| SGK3_NJ_F2 | CCGCCTCTTCTAAGGAGCTTT | T7/TIDE |
| SGK3_NJ_R2 | GAGTACTTCCCAAGTGCCTCC | T7/TIDE |
| 18S_qPCR_F | TGTGATGCCCTTAGATGTCC | Host cell gDNA qPCR |
| 18S_qPCR_R | TTATGACCCGCACTTACTGG | Host cell gDNA qPCR |
| Circ_qPCR_F | CCCTGCCGCAAGTTTATGCTGTAT | IE gene Circ cDNA qPCR |
| Circ_qPCR_R | GTAAGAAAGTCGTGCAGTGAATCGG | IE gene Circ cDNA qPCR |
| gB_qPCR_F | CACGGACCTGGTGGACAAGAAG | L gene gB cDNA qPCR |
| gB_qPCR_R | CTACCGTCACGTGCTGTGTAC | L gene gB cDNA qPCR |
| UL23_qPCR_F | CTCTGCTACCCCTTCGCCCGCTACT | E gene UL23 cDNA qPCR |
| UL23_qPCR_R | AGGGTGCACACGACGAGGTTGGC | E gene UL23 cDNA qPCR |
| PVRL2_qPCR_F | AGCCAGAAAGAATCTAAGGGCCAGG | PVRL2 gDNA qPCR |
| PVRL2_qPCR_R | CCCAGGTCGTCCGAATTATCATTCT | PVRL2 gDNA qPCR |
| flagPB5_F | ACAAAGACGATGACGACAAGtaaaaatgtcgacggtaccg | Amplify PiggyBac backbone |
| startPB3_R | catggtaaaccttcacgac | Amplify PiggyBac backbone |
| PB5gB_F | gtgtcgtgaaggtttaccatggccgctcgcggcggt | Amplify ORFs of BoHV-1 |
| PB3gB_R | CGTCATCGTCTTTGTAGTCtgcccccccgacgtcggc | Amplify ORFs of BoHV-1 |
| PB5gC_F | gtgtcgtgaaggtttaccatgggcccgctggggcgag | Amplify ORFs of BoHV-1 |
| PB3gC_R | CGTCATCGTCTTTGTAGTCcaggcgcgcccgggcctt | Amplify ORFs of BoHV-1 |
| PB5gD_F | gtgtcgtgaaggtttaccatgcaagggccgacattggccg | Amplify ORFs of BoHV-1 |
| PB3gD_R | CGTCATCGTCTTTGTAGTCcccgggcagcgcgctgtag | Amplify ORFs of BoHV-1 |
| PB5gE_F | gtgtcgtgaaggtttaccatgcaacccaccgcgccg | Amplify ORFs of BoHV-1 |
| PB3gE_R | CGTCATCGTCTTTGTAGTCgcggaggatggacttgagtcgcgc | Amplify ORFs of BoHV-1 |
| PB5gG_F | gtgtcgtgaaggtttaccatgcctgccgcccggacc | Amplify ORFs of BoHV-1 |
| PB3gG_R | CGTCATCGTCTTTGTAGTCgacgctgagcatcggctcgtacgc | Amplify ORFs of BoHV-1 |
| PB5gH_F | gtgtcgtgaaggtttaccatgcggcgcccgctctgc | Amplify ORFs of BoHV-1 |
| PB3gH_R | CGTCATCGTCTTTGTAGTCaaacaccgggaccgacgagtacccg | Amplify ORFs of BoHV-1 |
| PB5VP8_F | gtgtcgtgaaggtttaccatggacgccgctagggatgggc | Amplify ORFs of BoHV-1 |
| PB3VP8_R | CGTCATCGTCTTTGTAGTCgcggccgcccaggcgcgg | Amplify ORFs of BoHV-1 |

**Note:** iVT = *in vitro* transcription;

# Table E. Primers used for NextSeq

| **Primer** | **Sequence** |
| --- | --- |
| PCR1_F1 | TCGTCGGCAGCGTCAGATGTGTATAAGAGACAGTCTTGTGGAAAGGACGAAACACCG |
| PCR1_F2 | TCGTCGGCAGCGTCAGATGTGTATAAGAGACAGATCTTGTGGAAAGGACGAAACACCG |
| PCR1_F3 | TCGTCGGCAGCGTCAGATGTGTATAAGAGACAGGATCTTGTGGAAAGGACGAAACACCG |
| PCR1_F4 | TCGTCGGCAGCGTCAGATGTGTATAAGAGACAGCGATCTTGTGGAAAGGACGAAACACCG |
| PCR1_F5 | TCGTCGGCAGCGTCAGATGTGTATAAGAGACAGTCGATCTTGTGGAAAGGACGAAACACCG |
| PCR1_F6 | TCGTCGGCAGCGTCAGATGTGTATAAGAGACAGATCGATCTTGTGGAAAGGACGAAACACCG |
| PCR1_F7 | TCGTCGGCAGCGTCAGATGTGTATAAGAGACAGGATCGATCTTGTGGAAAGGACGAAACACCG |
| PCR1_F8 | TCGTCGGCAGCGTCAGATGTGTATAAGAGACAGCGATCGATCTTGTGGAAAGGACGAAACACCG |
| PCR1_F9 | TCGTCGGCAGCGTCAGATGTGTATAAGAGACAGACGATCGATCTTGTGGAAAGGACGAAACACCG |
| PCR1_F10 | TCGTCGGCAGCGTCAGATGTGTATAAGAGACAGTACGATCGATCTTGTGGAAAGGACGAAACACCG |
| PCR1_R | GTCTCGTGGGCTCGGAGATGTGTATAAGAGACAGCTAAAGCGCATGCTCCAGAC |
| idx_S502 | AATGATACGGCGACCACCGAGATCTACACCTCTCTATTCGTCGGCAGCGT*C |
| idx_S503 | AATGATACGGCGACCACCGAGATCTACACTATCCTCTTCGTCGGCAGCGT*C |
| idx_S504 | AATGATACGGCGACCACCGAGATCTACACAGAGTAGATCGTCGGCAGCGT*C |
| idx_S517 | AATGATACGGCGACCACCGAGATCTACACGCGTAAGATCGTCGGCAGCGT*C |
| idx_N701 | CAAGCAGAAGACGGCATACGAGATTCGCCTTAGTCTCGTGGGCTCG*G |
| idx_N702 | CAAGCAGAAGACGGCATACGAGATCTAGTACGGTCTCGTGGGCTCG*G |
| idx_N703 | CAAGCAGAAGACGGCATACGAGATTTCTGCCTGTCTCGTGGGCTCG*G |
| idx_N704 | CAAGCAGAAGACGGCATACGAGATGCTCAGGAGTCTCGTGGGCTCG*G |
| idx_N705 | CAAGCAGAAGACGGCATACGAGATAGGAGTCCGTCTCGTGGGCTCG*G |
| idx_N706 | CAAGCAGAAGACGGCATACGAGATCATGCCTAGTCTCGTGGGCTCG*G |

**Note:** 1^st^ PCR primers (PCR1_F1 to PCR1_R) were ordered as regular oligos and index primers (idx_S502 to idx_N706) as Ultramers from IDT with the last base (*) Phosphorothioated to prevent degradation. The barcodes are underlined.

# Table F. Reagents and consumables used in the study

| **Reagent** | **Supplier** | **Cat. I.D.** | **Purpose** |
| --- | --- | --- | --- |
| 2XHBS | VWR | J62623.AK | Lentivirus packaging |
| 2.5M CaCl_2_ | Jena Bioscience | BU-103-JEN | Lentivirus packaging |
| Lipofectamine 2000 | Thermo Fisher | 11668019 | transfection |
| DNase/RNase-Free Distilled Water | Life Technologies | 10977035 | Various |
| DMEM | Sigma | D5796 | Tissue culture |
| Horse serum | Sigma | H1138 | MBDK culture |
| Horse serum | Gibco | 26050088 | MBDK culture |
| Bovine Serum | Gibco | 10270 | HEK293FT culture |
| Fetal bovine serum | Gibco | 16170-078 | HEK293FT culture |
| Pen/Strep | Gibco | 15140122 | Cell culture |
| NEAA | Gibco | 11140035 | MBDK culture |
| Sodium Pyruvate | Gibco | 11360039 | MBDK culture |
| L-Glutamine | Gibco | 25030024 | MBDK culture |
| Trypsin solution | Sigma | T3924-100ML | Cell culture |
| BloodStor 100 DMSO | StemCell Technologies | 07951 | Cell freezing media |
| Blasticidin S HCl | Corning B.V. | 30-100-RB | Rosa26 targeting |
| Blasticidine S hydrochloride | Sigma | 15205-25mg | Rosa26 targeting |
| G418 | Sigma | G8168-10ML | HEK293FT culture |
| Puromycin Dihydrochloride | ThermoFisher | A1113803 | Library transduction |
| Saline, Buffered; HEPES; 1M | GE Hyclone | 10204932 | Library packaging |
| 150mm Petri dish | Corning | 10117320 | Library packaging |
| Syringe Filter, PES, 0.45um | Fisherbrand | 15216869 | Lentivirus packaging |
| 0.45um filter, 500ml | Corning | 430770 | Lentivirus filtration |
| Polybrene | Merck | TR-1003-G | Lentivirus transduction |
| NEB PCR Cloning Kit | NEB | E1203S | Construct cloning |
| Zero Blunt TOPO Cloning Kit | ThermoFisher | 450245 | PCR cloning |
| 10-betaStable Outgrowth Medium | NEB | B9035S | Library cloning |
| Ampicillin sodium salt | Sigma | A9518-25G | Library cloning |
| BbsI-HF | NEB | R3539S | Library cloning |
| BpiI(BbsI) (10U/uL) | Thermo Scientific | ER1011 | Library cloning |
| Quick Ligation Kit | NEB | M2200L | Library cloning |
| Adenosine 5’-Triphosphate (ATP) | NEB | P0756S | Library cloning |
| QIAquick Nucleotide Removal Kit | Qiagen | 28304 | Library cloning |
| HindIII-HF | NEB | R3104M | Genomic DNA digestion |
| NEBNext Ultra II Q5 Master Mix | NEB | M0544L | 2^nd^ PCR for NextSeq |
| 2X Q5 Hot Start Master Mix | NEB | M0494L | 1^st^ PCR for NextSeq |
| Gibson Assembly Kit | NEB | E5510S | To clone expression plasmids |
| 2X Phusion HF Master Mix | NEB | M0531L | PCR for T7 and Sanger seq |
| 2X DreamTaq Green PCR Master Mix | Thermo Scientific | K1081 | Routine PCR |
| PrimeSTAR GXL DNA polymerase | Takara Bio | R050A | Long Range PCR |
| NEBuilder HiFi DNA Assembly Cloning Kit | NEB | E5520S | Construct cloning |
| Plasmid plus Midi kit | Qiagen | 12943 | Plasmid prep |
| Plasmid plus Maxi kit | Qiagen | 12963 | Library plasmid prep |
| Qiaprep Spin Miniprep kit | Qiagen | 27104 | Plasmid preparation |
| NucleoSpin Blood L Column | Machery-Nagel | 12761021 | Genomic DNA isolation |
| NucleoSpin Blood XL Column | Machery-Nagel | 12731021 | Genomic DNA isolation |
| Ribonuclease A | Sigma | R6513-1G | Genomic DNA isolation |
| DNeasy Blood & Tissue kit | Qiagen | 69504 | Genomic DNA isolation |
| Quick-DNA Microprep kit | Zymo Research | D3020 | Genomic DNA isolation |
| Quick-DNA Miniprep Plus Kit | Zymo Research | D4068 | Genomic DNA isolation |
| Qubit dsDNA HS Assay Kit | Invitrogen | Q32854 | DNA quantification |
| QuickExtract DNA Extraction Solution | Lucigen | QE09050 | Genomic DNA extraction |
| 10-beta Electrocompetent E Coli | NEB | C3020K | Library cloning |
| Gene Pulser/MicroPulser Cuvettes, 0.1cm gap | Bio-rad | 1652089 | Library cloning |
| NEB Stable Competent Cells | NEB | C3040I | Construct cloning |
| Top10 Competent Cells | Invitrogen | C404003 | Plasmid transformation |
| Agencourt AMPure XP | Beckman Coulter | A63880 | PCR cleanup for NextSeq |
| Agarose UltraPure | Life Technologies | 16500500 | DNA/RNA electrophoresis |
| ChargeSwitch PCR Clean-up kit | Invitrogen | CS12000 | PCR cleanup for Sanger Seq |
| Zymoclean Gel DNA Recovery | Zymo Research | D4001 | Library cloning |
| NucleoSpin Gel and PCR Clean-up Kit | Machery-Nagel | 11992242 | Gel extraction and PCR cleanup |
| MinElute PCR Purification Kit | Qiagen | 28004 | PCR cleanup |
| Neon Transfection System 100 uL Kit | ThermoFisher | MPK10025 | MDBK transfection |
| T7 Endonuclease I | NEB | M0302S | Gene editing detection |
| HiScribe T7 Quick High Yield RNA synthesis kit | NEB | E2050s | sgRNA *in vitro* transcription |
| Hiscribe T7 ARCA mRNA Kit | NEB | E2060S | mRNA *in vitro* transcription |
| RNASecure RNase Inactivation Reagent | ThermoFisher | AM7005 | RNA treatment |
| RNeasy mini Kit | Qiagen | 74104 | RNA isolation and cleanup |
| Doxycycline hyclate | Sigma | D9891-1G | dCas9 induction |
| Avicel RC/CL | FMC Biopolymer | RC-581 | Plaque assays |
| 10% Neutral Buffered Formalin | CellPath | BAF-0010-20A | Plaque assays |
| Toluidine Blue | Sigma | T3260 - 100G | Plaque assays |
| [LightCycler 480 SYBR Green I Master](https://shop.roche.com/shop/store/ProductDisplay?catalogId=10001&partNumber=3.5.8.1.1.2) | Roche | 04887352001 | qPCR |
| TRIzol LS reagent | Thermo Fisher | 10296028 | RNA extraction |
| Turbo DNA-free kit | Invitrogen | AM1907 | Residual DNA removal from RNA sample |
| Mouse anti -GFP antibody (JL-8) | Takara bio | 632381 | Detection of VP26-GFP by WB |
| Rabbit anti beta-actin antibody | Invitrogen | MA5-32540 | Targeting loading control protein beta-actin |
| Rabbit anti FLAG antibody | Sigma | F7425 | Western blot |
| Licor Donkey anti-mouse Dylight 680 Ab | Licor | 926-68072 | Western blot |
| Licor Donkey anti-rabbit DyLight 800 Ab | Licor | 926-32213 | Western blot |
| Alexa 594 conjugated a-mouse secondary Ab | Thermo | A-11005 | Immunofluorescent staining |
| Alexa 647 conjugated a-rabbit secondary Ab | Thermo | A-21245 | Immunofluorescent staining |

# References

1. Tzelepis K, Koike-Yusa H, De Braekeleer E, Li Y, Metzakopian E, Dovey OM, et al. A CRISPR Dropout Screen Identifies Genetic Vulnerabilities and Therapeutic Targets in Acute Myeloid Leukemia. Cell Reports. 2016 Oct 18;17(4):1193–205.

2. Protocol for Lentivial Vector (LV) Production (2 nd Generation Packaging).

3. Cermak T, Doyle EL, Christian M, Wang L, Zhang Y, Schmidt C, et al. Efficient design and assembly of custom TALEN and other TAL effector-based constructs for DNA targeting.

4. Tan W, Carlson DF, Lancto CA, Garbe JR, Webster DA, Hackett PB, et al. Efficient nonmeiotic allele introgression in livestock using custom endonucleases. Proceedings of the National Academy of Sciences of the United States of America. 2013 Oct 8;110(41):16526–31.

5. Fink M, Flekna G, Ludwig A, Heimbucher T, Czerny T. Improved translation efficiency of injected mRNA during early embryonic development. Developmental Dynamics. 2006 Dec 1;235(12):3370–8.

6. Joung J, Konermann S, Gootenberg JS, Abudayyeh OO, Platt RJ, Brigham MD, et al. Genome-scale CRISPR-Cas9 knockout and transcriptional activation screening. Nature Protocols. 2017 Apr 1;12(4):828–63.

7. Desjardins P, Conklin D. NanoDrop microvolume quantitation of nucleic acids. Journal of Visualized Experiments. 2010;(45).

8. Brinkman EK, Chen T, Amendola M, Van Steensel B. Easy quantitative assessment of genome editing by sequence trace decomposition. Nucleic Acids Research. 2014;42:168.

9. Li W, Xu H, Xiao T, Cong L, Love MI, Zhang F, et al. MAGeCK enables robust identification of essential genes from genome-scale CRISPR/Cas9 knockout screens. Genome Biology. 2014 Dec 5;15(12):554.

1. ^†^ † Deceased [↑](#footnote-ref-1)
