# Supplementary material for: GARP and EARP are required for efficient BoHV-1 replication as identified by a genome wide CRISPR knockout screen
Source: PLoS Pathog. 2023 Dec 6;19(12):e1011822. doi: 10.1371/journal.ppat.1011822 (PMC10727446; doi:10.1371/journal.ppat.1011822)
Supplement: S2 Text — Table A. Numbers of genes targeted, and guides included in the btCRISPRko.v1 library. Table B. Libraries produced from this study. Table C. Number of cells recovered from 1st screen. Table D. Number of cells recovered from 2nd screen. Table E. Knockout clone genotypes. Fig A. Knock-in (KI) of Cas9 to rosa26. A. Knock-in and genotyping strategies. A genome editor designed to target first intron of rosa26 generates a double strand break and a plasmid with a EF1a promoter driven Cas9 expression cassette flanked by homology sequences (orange colored) is used to repair the break, incorporating the expression cassette. To identify targeted clones, PCR primer set F+R binding outside the homology arms (horizontal read arrows) is used for genotyping. B. TALEN mRNA pair TAL1.6, chosen out of six editors tested (T7E1 results shown on gel), cuts in intron 1 and was co-transfected with HDR plasmid expressing the Cas9-2a-Blasticidin cassette. Single cell clones were isolated and genotyped. C. Genotyping results. PCR amplicons from the 4kb Wild Type (wt, filled triangles) and 11kb targeted (tg, filled triangle) alleles are indicated. Homozygote clones are labelled as +/+. Fig B. Lentivirus transduction efficiency is low in wt MDBK cells. A. bright field visualization of HEK293FT cells 48 hours after being transfected with the library plasmid pool, pMD.2 and psPAX2 for serum-free lenti-virus packing. B. Same cells under the BFP filter showing strong BFP expression, indicating efficient transfection and packaging. C. CRISPR library transduction in HEK293FT cells. D. CRISPR library transduction in Cas9+/+ cells without TRIM5 KO with four times the virus as that used on HEK293FTs. Fig C. Plaque assays to compare replication of BoHV-1 in wild type MDBK cells and homozygous Cas9 clones. A. Plaque sizes measurements from wt, and three Cas9+/+ clones infected with GFP tagged BoHV-1 virus, repeat n = 3 (n.s.: not significant with p-value>0.05 based on a two-tailed t-test). B. Plaque number [file ppat.1011822.s009.docx]

**GARP and EARP are required for efficient BoHV-1 replication as identified by a genome wide CRISPR knockout screen**

Wenfang S. Tan^1,*^, Enguang Rong^1,^ ^†^ ^[[1]](#footnote-1)^, Inga Dry^1^, Simon G. Lillico^2,4^, Andy Law^3^, Paul Digard^1^, Bruce Whitelaw^2,4^, Robert G. Dalziel^1^

1. Division of Infection and Immunity, University of Edinburgh, Edinburgh, Scotland, United Kingdom

2. Division of Functional Genetics and Development, University of Edinburgh, Edinburgh, Scotland, United Kingdom

3. Division of Genetics and Genomics, University of Edinburgh, Edinburgh, Scotland, United Kingdom

4. Centre for Tropical Livestock Genetics and Health, the Roslin Institute, Easter Bush Campus, University of Edinburgh, , Edinburgh, Scotland, United Kingdom

* [wtan2@exseed.ed.ac.uk](mailto:wtan2@exseed.ed.ac.uk)

**Keywords:** CRISPR/Cas9, knockout screen, cattle, BoHV-1, GARP, EARP

**Supplementary data**

**References**

Table of Contents

[Fig A. Knock-in of EF1a-Cas9 to both alleles of the rosa26 locus 3](#_Toc152082859)

[Fig B. Lentivirus transduction efficiency in wt MDBK cells is very low 4](#_Toc152082860)

[Fig C. Cas9 expression does not affect BoHV-1 infection in MDBKs 5](#_Toc152082861)

[Fig D. The loss of TRIM5a does not affect BoHV-1 infection in MDBKs 6](#_Toc152082862)

[Fig E. Plaque assay to compare BoHV-1 replication in Cas9+/+ cells and Cas9+/+; TRIM5-/- clones 7](#_Toc152082863)

[Fig F. Library design validation 7](#_Toc152082864)

[Fig G. Cloning, packaging, and transducing lentiviral libraries into Cas9+/+;TRIM5-/- MDBKs 8](#_Toc152082865)

[Fig H. NGS and pairwise comparisons to assess library performance and identify candidate host genes 9](#_Toc152082866)

[Fig I. Quality control and performance comparison between the lentiviral g2 and g5 libraries 10](#_Toc152082867)

[Fig J. Library performance and specificity comparison 10](#_Toc152082868)

[Fig K. PiggyBac CRISPR Knockout libraries 11](#_Toc152082869)

[Fig L. GFP tagged BoHV-1 virus replicates in MDBKs 11](#_Toc152082870)

[Fig M. FACS sort to isolate sub-populations with varied intensities of GFP 12](#_Toc152082871)

[Fig N. GO analysis of candidates 13](#_Toc152082872)

[Fig O. CRISPR screen identifies pro-viral genes in GFP Neg populations. 14](#_Toc152082873)

[Fig P. Candidates identified in the GFP low sub-populations 14](#_Toc152082874)

[Fig Q. Sizes of plaques of viruses grown in GARP/EARP knockout and rescue cells. 15](#_Toc152082875)

[Fig R. Plaque size in VPS50KO and VPS54KO cells. 15](#_Toc152082876)

[Fig S. An antibody against VP8 of BoHV-1 16](#_Toc152082877)

[Table A. Numbers of genes targeted, and guides included in the btCRISPRko.v1 library 18](#_Toc152082878)

[Table B. Libraries produced from this study 18](#_Toc152082879)

[Table C. Number of cells recovered from 1^st^ screen 18](#_Toc152082880)

[Table D. Number of cells recovered from 2^nd^ screen 18](#_Toc152082881)

[Table E. Knockout clone genotypes 19](#_Toc152082882)

[References 20](#_Toc152082883)

#

# Fig A. Knock-in of EF1a-Cas9 to both alleles of the rosa26 locus


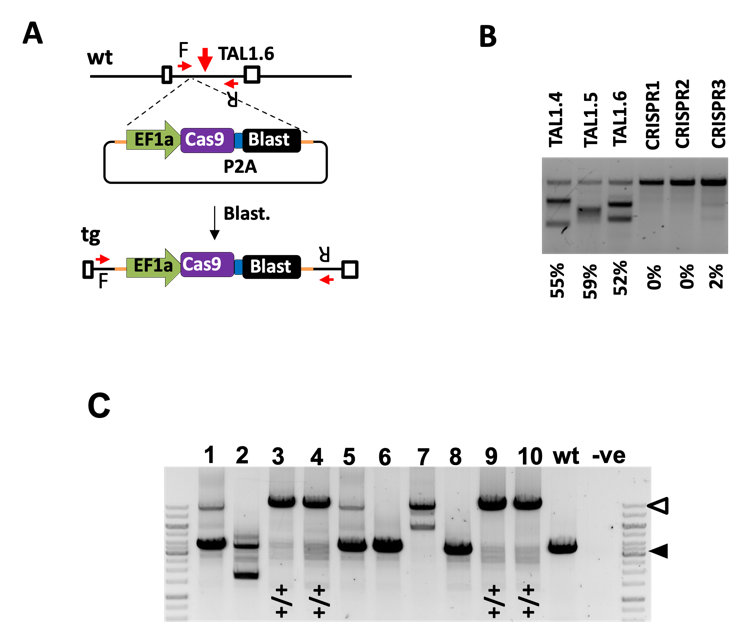


**Fig A.** Knock-in (KI) of Cas9 to rosa26. **(a).** Knock-in and genotyping strategies. A genome editor designed to target first intron of rosa26 generates a double strand break and a plasmid with a EF1a promoter driven Cas9 expression cassette flanked by homology sequences (orange colored) is used to repair the break, incorporating the expression cassette. To identify targeted clones, PCR primer set F+R binding outside the homology arms (horizontal read arrows) is used for genotyping. **(b).** TALEN mRNA pair TAL1.6, chosen out of six editors tested (T7E1 results shown on gel), cuts in intron 1 and was co-transfected with HDR plasmid expressing the Cas9-2a-Blasticidin cassette. Single cell clones were isolated and genotyped. **(c).** Genotyping results. PCR amplicons from the 4kb Wild Type (wt, filled triangles) and 11kb targeted (tg, filled triangle) alleles are indicated. Homozygote clones are labelled as +/+.

# Fig B. Lentivirus transduction efficiency in wt MDBK cells is very low

Compared to HEK293FT cells, MDBKs are ~30-60 times less transducible (**Fig BC and BD**). Although this is sufficient for single gene knockouts with our efficient serum free lentivirus packaging protocol (**Fig BA and BB**), it becomes labour intensive and costly to deliver a genome wide CRISPR library, as it requires large quantities and high titres of lentivirus stocks to achieve sufficient coverage.


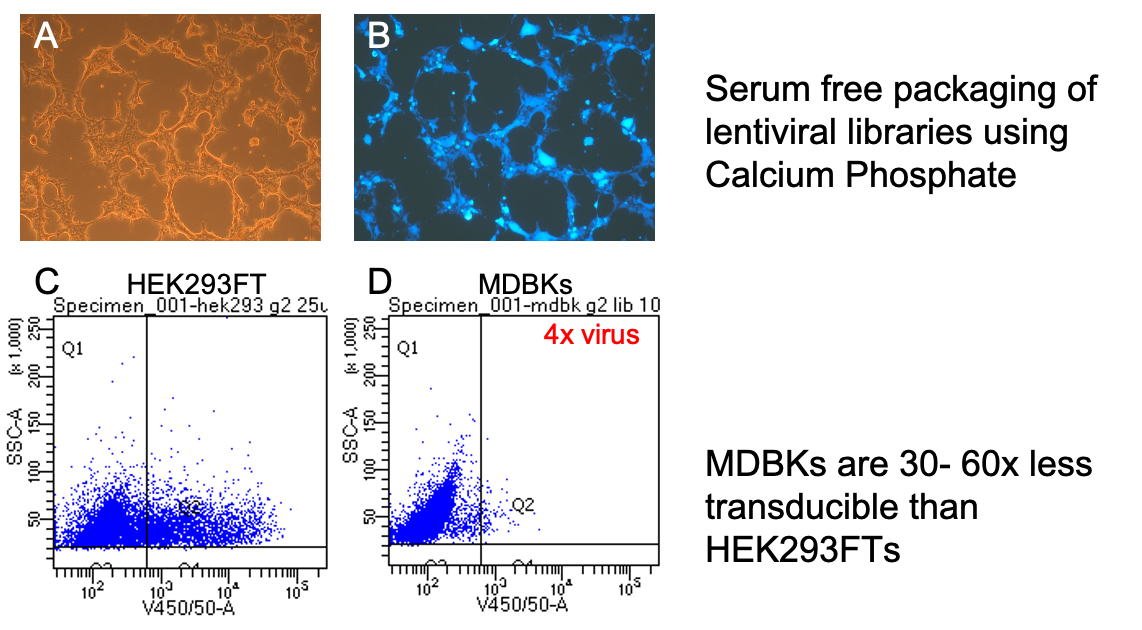


**Fig B. Lentivirus transduction efficiency is low in wt MDBK cells.** (a). bright field visualization of HEK293FT cells 48 hours after being transfected with the library plasmid pool, pMD.2 and psPAX2 for serum-free lenti-virus packing. (b). Same cells under the BFP filter showing strong BFP expression, indicating efficient transfection and packaging. (c). CRISPR library transduction in HEK293FT cells. (d). CRISPR library transduction in Cas9+/+ cells without TRIM5 KO with four times the virus as that used on HEK293FTs.

# Fig C. Cas9 expression does not affect BoHV-1 infection in MDBKs

To decide whether Cas9 expression would affect BoHV-1 replication, we conducted plaque assays in Cas9+/+ MDBKs and wt MDBK cells by infection with the GFP tagged BoHV-1 virus. We compared the sizes (**Fig CA**) and numbers (**Fig CB**) of plaques formed in the three homozygous Cas9 clones, #C, #P and #AH and those formed in the wt MDBK cells. No significant difference between the wt MDBKs and Cas9 clones was observed (p>0.05), indicating that Cas9 expression has no effect on BoHV-1 replication in MDBK cells.

**
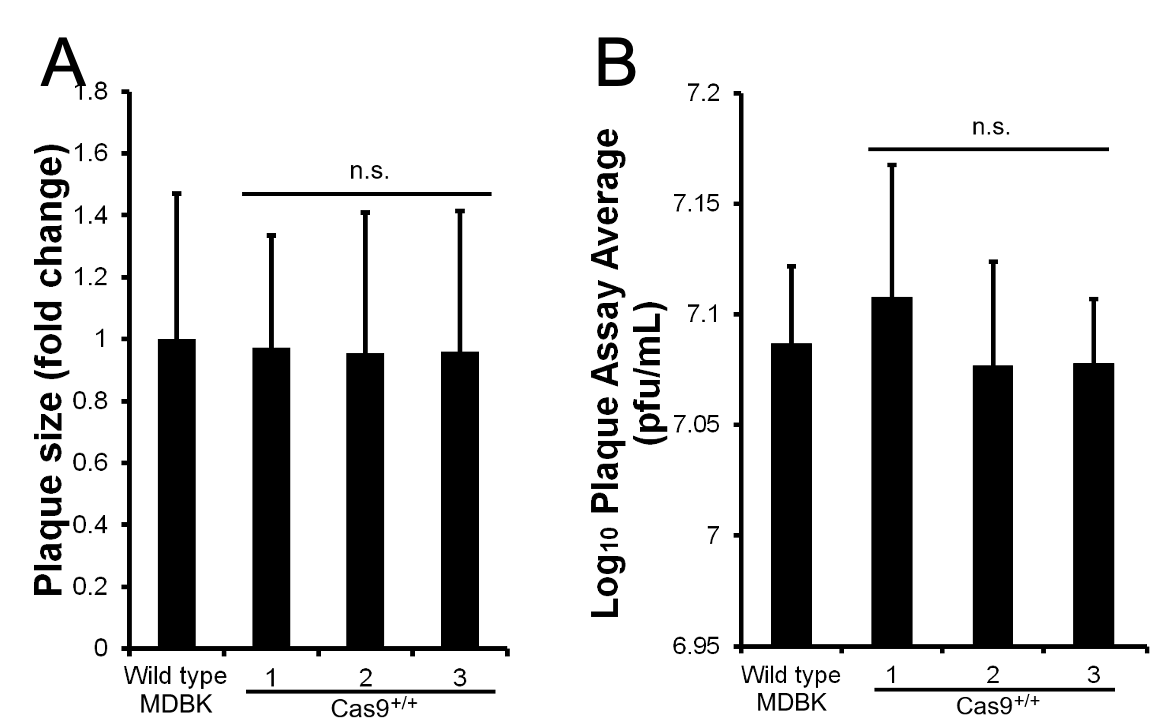
**

**Fig C. Plaque assays to compare replication of BoHV-1 in wild type MDBK cells and homozygous Cas9 clones.** (a). Plaque sizes measurements from wt, and three Cas9^+/+^ clones infected with GFP tagged BoHV-1 virus, repeat n=3 (n.s.: not significant with p-value>0.05 based on a two-tailed t-test). (b). Plaque number counts calculated as pfu/mL from wt, and the Cas9^+/+^ clones.

# Fig D. The loss of TRIM5a does not affect BoHV-1 infection in MDBKs

To compare BoHV-1 replication in wild type MDBKs and TRIM5a knockout clones, we conducted plaque assays using the GFP tagged BoHV-1 virus (**Figs D and L**). We studied the numbers (**Fig DB**) and sizes (**Fig DA**) of plaques formed in wild type MDBKs and two TRIM5-/- clones, A44 and B13. No significant difference between the wt MDBKs and KO clones was evident by student’s t-test comparisons (p>0.05). We also compared the Cas9+/+ clones to Cas9+/+; TRIM5 -/- clones in terms of plaque sizes and numbers and no difference was observed either (**Fig D**), indicating that the loss of TRIM5a has no effect on BoHV-1 replication in MDBK cells.

**
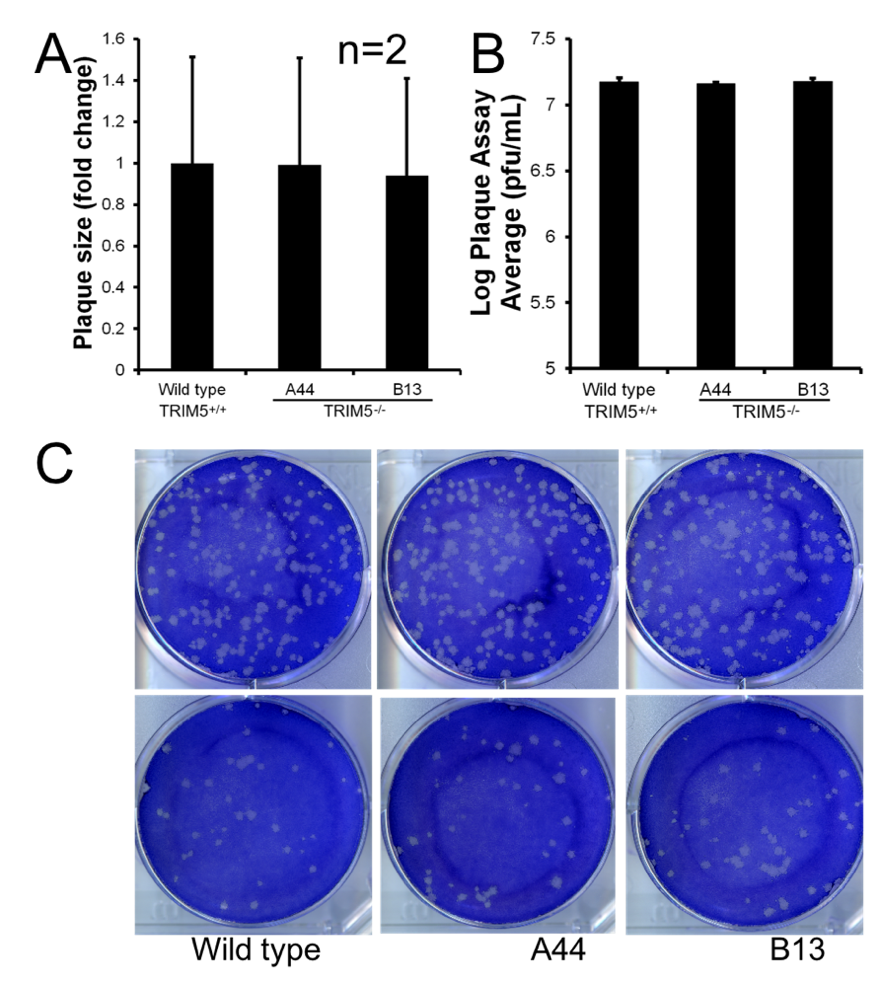
**

**Fig D. Plaque assays to compare replication of BoHV-1 in wild type MDBK cells and TRIM5-/- clones.** (a). Plaque sizes measurements from wt, and two TRIM5 KO clones, A44 and B13 infected with GFP tagged BoHV-1 virus, repeat n=2. (b). Plaque number counts calculated as pfu/mL from wt, and the TRIM5 KO clones. (c). Sample plaque assay results with the same quantities of virus.

# Fig E. Plaque assay to compare BoHV-1 replication in Cas9+/+ cells and Cas9+/+; TRIM5-/- clones

**
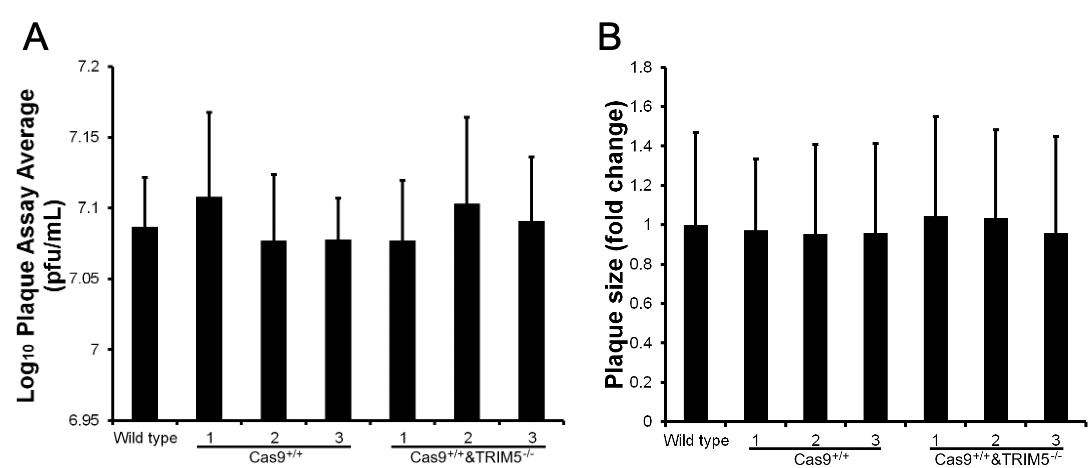
**

**Fig E. Plaque assay to compare BoHV-1 replication in Cas9+/+ only cells and Cas9+/+; TRIM5-/- clones.** (a). Titer of virus grown in Cas9+/+ or Cas9+/+;TRIM5-/- cells; (b). Average size of plaques grown. All comparisons conducted by single factor ANOVA results non-significant results.

# Fig F. Library design validation

Prior to library cloning, its design was validated by testing editing efficiency of guides against a list of genes. Guides were picked randomly from the library for chosen genes and cloned into lentiGuide-Puro or PB_U6gRNA2-CAGpuro for lentiviral or PiggyBac based delivery into Cas9+/+ MDBKs. Alternatively, some guides were *in vitro* transcribed into sgRNA and transfected into MDBKs for leave-no-trace editing. After four days of Puromycin selection or 2-3 days of recovery after transfection with sgRNA, the genomic DNA was harvested for T7 assays and TIDE analysis to determine CRISPR cutting efficiency. Reassuringly, all guides tested are functional with editing efficiency ranging from 11.4% to 79% (**Fig F**). And regardless of method, PB and lentivirus delivered similar cutting efficiency (**Fig FA**). *In vitro* transcribed sgRNA also mediated efficient editing with up to 65% gene editing (**Fig FB**); this selection-marker-free and leave-no-trace editing method is very convenient for generating gene knockout clones.

**
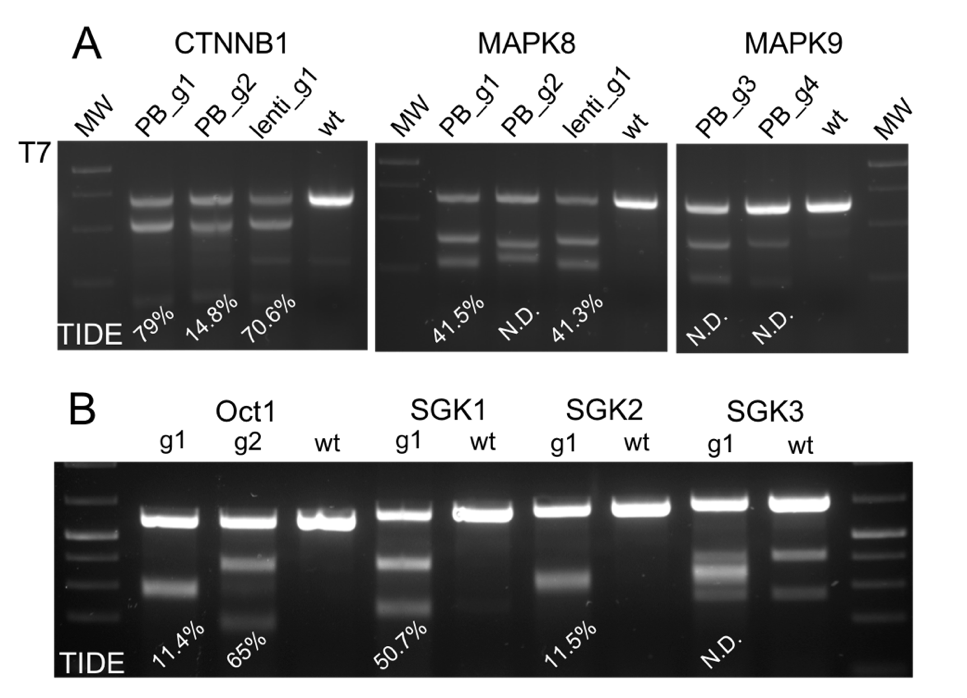
**

**Fig F. Testing CRISPRs designed for the CRISPRko library in MDBK cells.** (a). Cutting efficiency of guides delivered by lentivirus or PiggyBac targeting CTNNB1, MAPK8 and MAPK9. (b). Cutting efficiency of guides *in vitro* transcribed as sgRNA and delivered by transfection, targeting Oct1, SGK1, SGK2, and SGK3.

# Fig G. Cloning, packaging, and transducing lentiviral libraries into Cas9+/+;TRIM5-/- MDBKs

We produced four genome wide CRISPR knockout libraries for cattle using the same oligo pool (**Table B**), K2g2, K2g5 (a.k.a btCRISPRko.v1, S1 **Data**), PBg2 and PBg5, based on different sgRNA scaffolds (g2 and g5) and delivery methods (lentivirus and piggyBac Chen *et al.* (1) developed an optimized sgRNA scaffold that was shown to mediate higher CRISPR cutting activity, by removing the potential premature T7 polymerase stop signal and extending the hairpin stem loops for better stability on the target. This has been confirmed in human and mouse cell lines in a few small- and large-scale studies(2,3). To determine which scaffold we should adopt for our screen, we generated two lentivirus libraries based on these scaffolds (**Figs I and J**) and compared their performances in the Cas9 +/+; TRIM5-/- cells (**Fig 2**). In addition, to make the btCRISPRko.v1 broadly applicable, the CRISPRs were also cloned into PB-U6gRNA(BbsI)-PGKpuro2ABFP and PB-U6gRNA5(BbsI)-PGKpuro2ABFP-W, to generate two additional libraries, PBg2btCRISPRko.v1 and PBg5btCRISPRko.v1 for delivery by transfection and transposition. This approach could facilitate the use of genome wide CRISPR knock out screens in hard to transduce cell types such as primary macrophages.

**
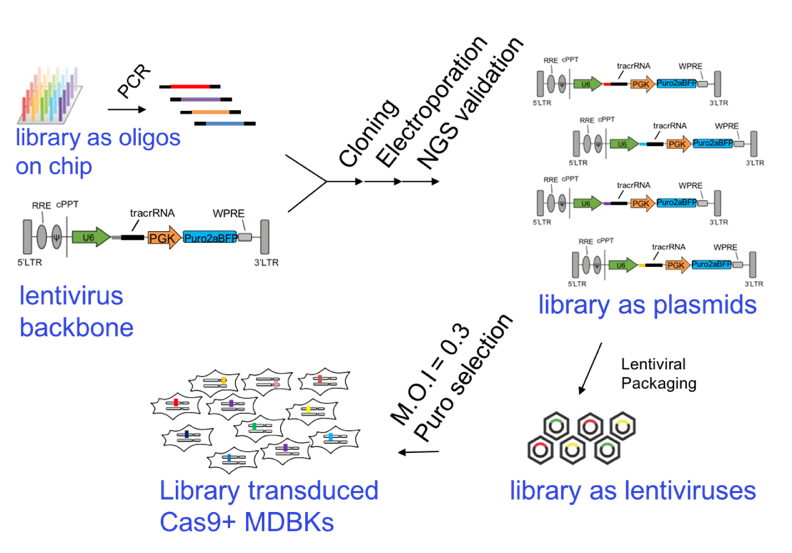
**

**Fig G. Stepwise CRISPR library cloning, packaging, and transduction. The l**ibrary was synthesized as a pool of oligos and PCR amplified to convert to dsDNA. The fragments containing CRISPRs were then ligated into either lentivirus (illustrated as example) or piggyBac vectors that contain a hU6 promoter to drive sgRNA expression and a Puro2aBFP marker for Puromycin selection. If using lentiviral delivery, the library was packaged as lentivirus and transduced into Cas9 expressing cells at low MOI to produce library expressing cells with single sgRNA integrations.

# Fig H. NGS and pairwise comparisons to assess library performance and identify candidate host genes

By PCR and next generation sequencing, we obtained copy numbers of guides in all the samples collected from our screen (S2 and S3 **Data files**). Pairwise comparisons between samples can identify enriched or depleted guides, and depletion or enrichment of corresponding genes. To illustration, guide RNA copy numbers are compared between the GFP Negative and GFP High sub-populations, enrichment of guides in the Negative sample relative to the High leads to depletion of genes targeted by these guides in the Negative sample, and identification of essential or pro-viral host genes for BoHV-1 infection. On the contrary, depletion of guides in the Negative sample leads to enrichment of genes that can be anti-viral.


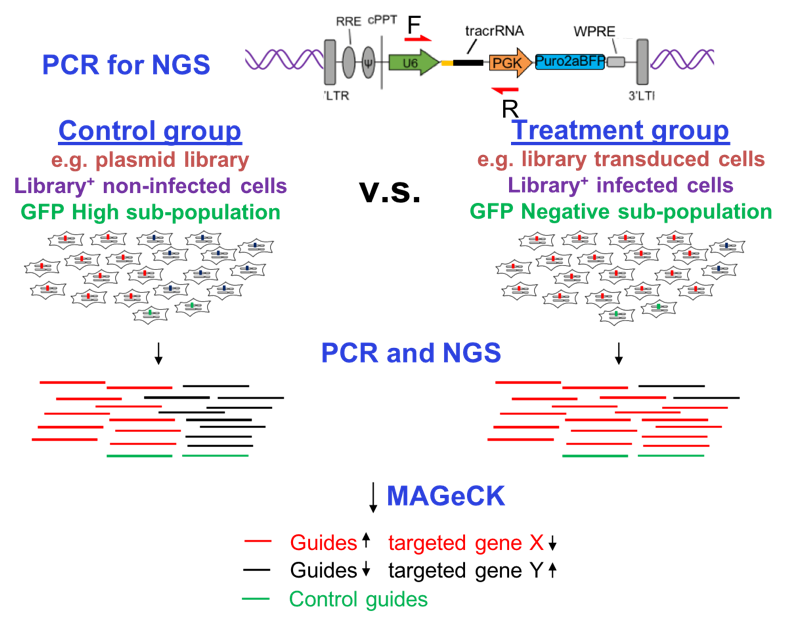


**Fig H. Next generation sequencing of screening samples to identify candidate genes with depleted or enriched guide RNA.** To determine copy numbers of guides, PCR using primers PCR1_Fx + PCR1_R (red arrows, Table E) are used to amplify the fragments containing CRISPR sequences. PCR products from different samples are then barcoded using a 2^nd^ PCR with a unique combination of indexes and sequenced as a pool. Comparison of guide RNA copy numbers between samples is conducted by MAGeCK to identify enriched or depleted guides and their targeted genes.

# Fig I. Quality control and performance comparison between the lentiviral g2 and g5 libraries

To examine guide RNA distribution and accuracy, the two lentiviral libraries were sequenced by NextSeq at 40x sequencing depth (**S1 Data**). After read processing and counting all guides included in the libraries, we observed good distribution and presence of the majority of the guides for both libraries (99.6% vs 98.8%). However, the g5 library is slightly less uniform than g2, likely due to PCR artefacts during library cloning. After deciding on using the lentiviral g5 library for our screening, we re-sequenced the K2g5 library with higher sequencing depth, at 200x together with library transduced cells prior to our BoHV-1 screen (S2 **Data**).

**
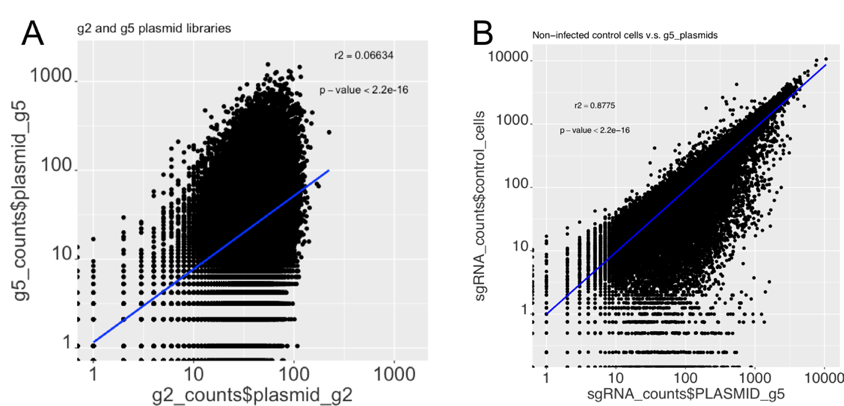
**

**Fig I. NextSeq of the CRISPR libraries to examine CRISPR copy number distribution.** (a). Sequencing of the K2g2 and K2g5 plasmid libraries at 40X sequencing depth. (b). Sequencing of the K2g5 library and library transduced Cas9+/+; TRIM5-/- cells at 200X sequencing depth.

# Fig J. Library performance and specificity comparison

By converging data from several CRISPR screening studies, Hart *et al.* identified a list of 684 core essential genes (CEG2.0) shared among 17 genome wide CRISPR knockout screens in human cell lines(4). The CEGs can be used as good indicators of library performance; since if a CEG targeting CRISPR works well, it should be gradually removed from the cell population during extended culture. Thus, a collective drop out of CEG2.0 targeting guides indicates library functionality; and the bigger the shift, the better the performance. To compare performance of the two lentivirus libraries, we packaged, titrated, and transduced them into MDBK cells using the same protocols. After seven days of Puromycin selection, genomic DNA were harvested from the two cell populations and the CRISPR regions were PCR amplified and sequenced by NextSeq at 40x depth (**Fig H and S1 Data**). We examined the degrees of drop out of CEG2.0 targeting guides in the two MDBK populations relative to the plasmid libraries and saw a much bigger shift in g5 transduced cells compared to g2, indicating better performance of g5 than g2 (**Fig J**). For both libraries, we observed relative stable or unchanged distribution of guides targeting non-essential genes and non-cutting control guides, indicating good overall specificity and control of off targeting from both libraries. Based on these results, we decided to use g5 library transduced cells for our BoHV-1 screens.

**
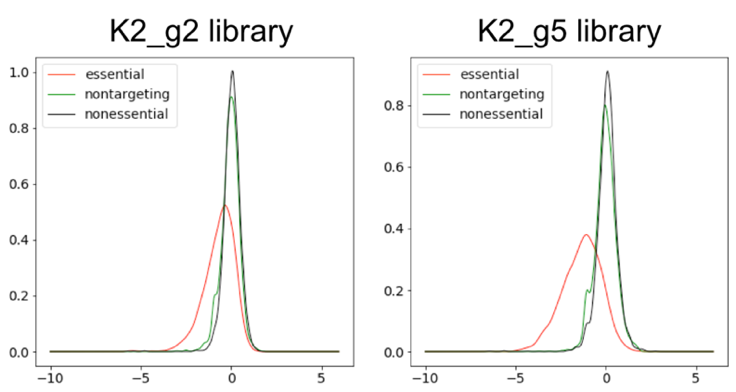
**

**Fig J. Library performance and specificity comparison between the K2g2 and K2g5 libraries.** Plots show log2 fold changes in copy numbers of guides targeting essential genes(red), non-essential genes(black), and non-targeting control guides(green) in the two cell populations transduced with the K2g2 or K2g5 library compared to the plasmids. The data was plotted using a python script adapted from Hart et al. 2017(4).

# Fig K. PiggyBac CRISPR Knockout libraries

The cloning of these PiggyBac libraries underwent the same quality control procedures as the lentiviral libraries, with above 1,000x depth, below 0.3% background and above 90% accuracy. A colony formation assay was conducted to test whether the libraries could transpose efficiently. When a plasmid expressing the piggyBac transposase i.e. hypBase(5) was added in a pilot library transfection and selection experiment using Cas9 expressing MDBKs (**Fig 2**), we obtained significantly more clones after puromycin selection compared to cells without transposase, indicating efficient CRISPR library integration by transposition.

**
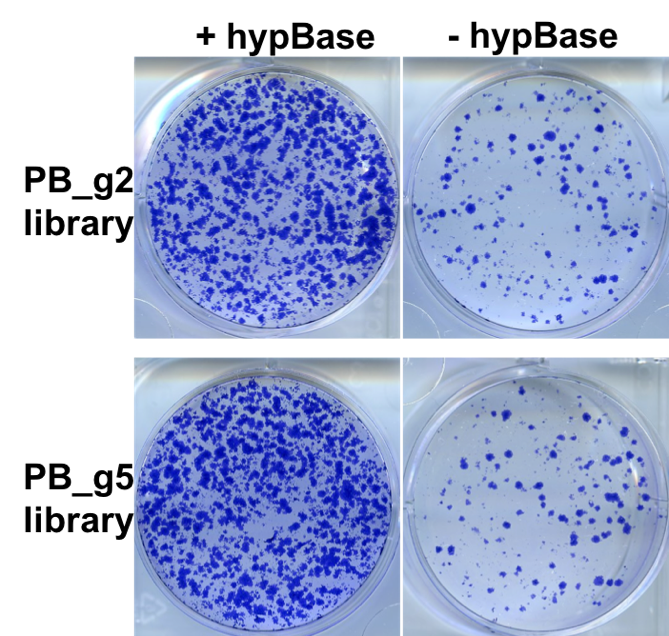
**

**Fig K. PiggyBac transposition mediated CRISPR library delivery into Cas9+/+ MDBK cells.** Cas9+/+ transfected with either library with (left panels) or without (right panels) transposase hypBase and selected with Puromycin to test library transposability. After Puro selection, cells were stained with Giemsa for colony visualization.

# Fig L. GFP tagged BoHV-1 virus replicates in MDBKs


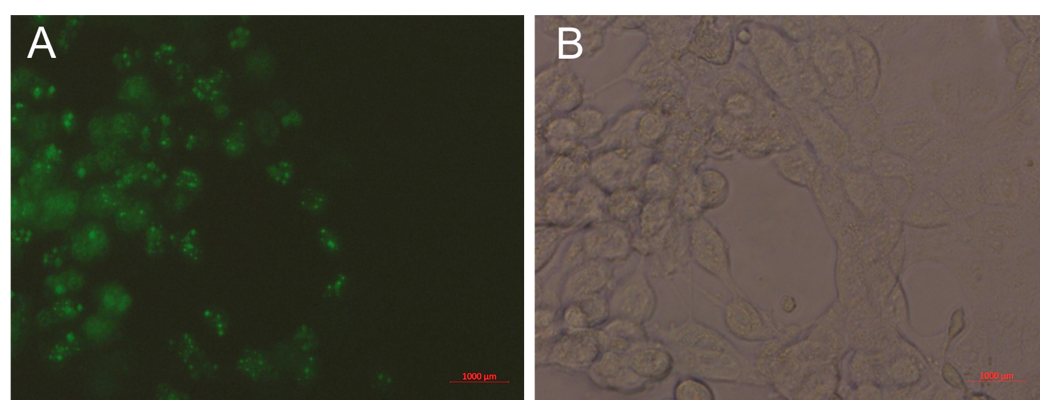


**Fig L. GFP tagged BoHV-1 virus infecting wt MDBK cells.** (a). 8 hours post infection of MDBKs by the GFP tagged virus. (b). image of the same cells under bright field.

# Fig M. FACS sort to isolate sub-populations with varied intensities of GFP

For the 1^st^ screen, 9.5-10 hours post infection of library transduced TRIM5-/-; Cas9+/+ MDBKs at MOI=2, cells were harvested for FACS sorting to isolate sub-populations with different levels of BoHV-1 infection based on GFP intensity :Negative, Low, Medium, and High (**Fig L**). The gating for the Negative was based on the non-infected cells, and the Low, Medium and High gates have equal width across the spread of the rest of the cells. Genomic DNA was then isolated from these sub-populations, PCR amplified and sequenced by Illumina NextSeq to examine distribution of guides (**Fig** H and S2 **Data**). For the 2^nd^ screen, cells were infected the same as the 1^st^ screen but harvested at 8 h.p.i. The gatings were set to collect all GFP Negative cells based on the non-infected cells and ~10% of live cells per sub-population for GFP Low, Medium and High with substantial space between gates. Cells were then processed and sequenced as per the 1^st^ screen.

**
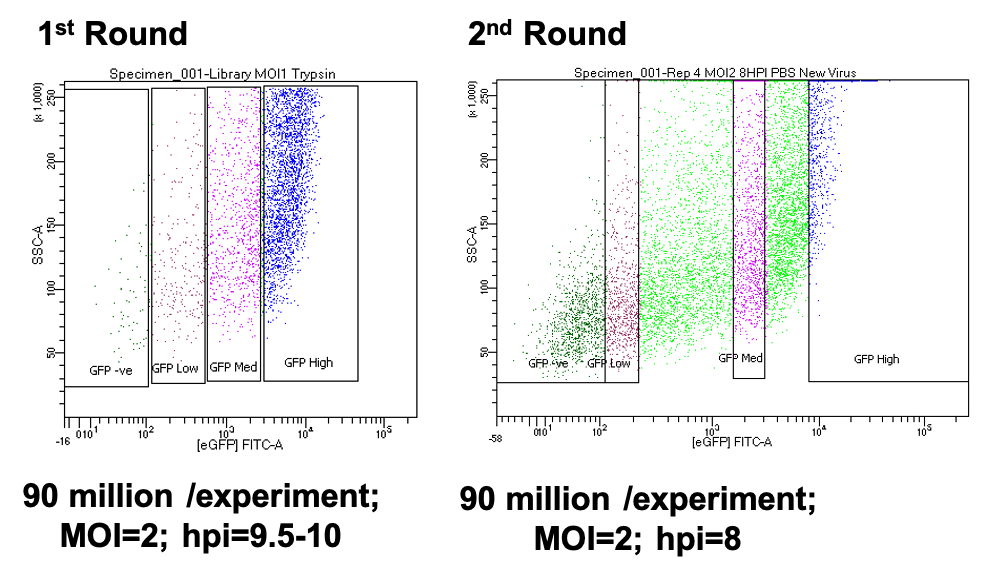
**

**Fig M. FACS sort to collect sub-populations of different degrees of BoHV-1 replication.** FACS plots with four gatings to collect sub-populations of live cells with GFP Negative, GFP Low, GFP Medium, and GFP High signals from 1^st^ or 2^nd^ round of screen.

# Fig N. GO analysis of candidates

**
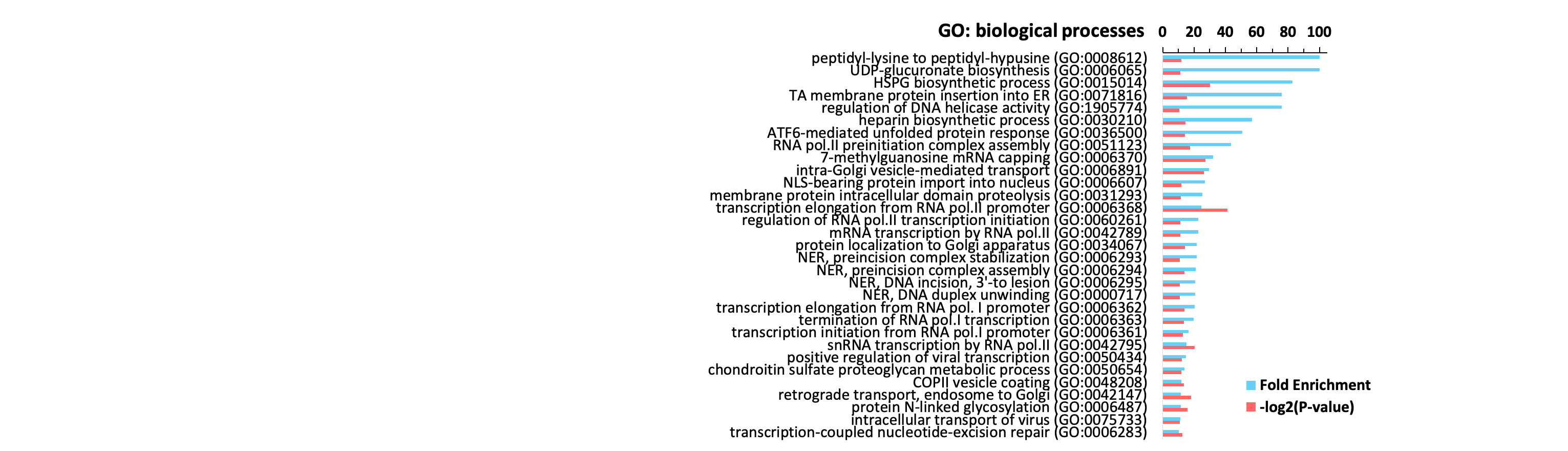
**

**Fig N. Gene Ontology analysis of pro-viral candidates identified by the screen.** GO reveals the top biological processes that can be important for the virus using the Fold Enrichment >=10, and -log2(p-value)>=10 statistical cut-offs (To see the full list, please refer to S6 **Data**).

# Fig O. CRISPR screen identifies pro-viral genes in GFP Neg populations.

**
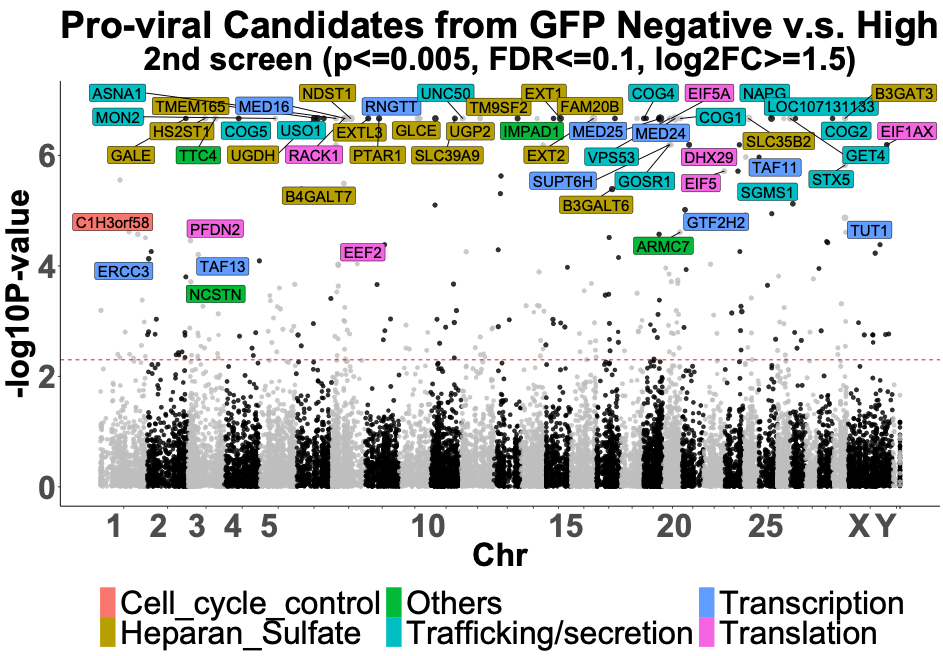
**

**Fig O. Candidates identified in the GFP Negative sub-population.** Data shown is from 2^nd^ screen, labels only applying to pro-viral candidates with stringent cut-offs: p<0.005, FDR<=0.1, and log2fc>=1.5 (S3 Data).

# Fig P. Candidates identified in the GFP low sub-populations

**
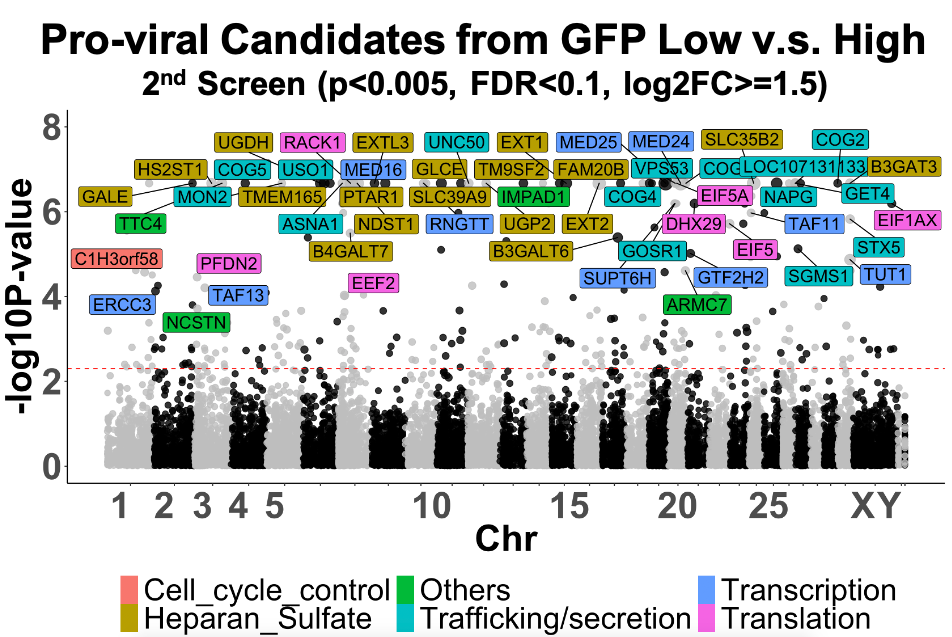
**

**Fig P. Candidates identified in the GFP Low sub-population.** Data shown is from 2^nd^ screen, labels only applying to pro-viral candidates with stringent cut-offs: p<0.005, FDR<=0.1, and log2fc>=1.5 (**S3 Data**).

# Fig Q. Sizes of plaques of viruses grown in GARP/EARP knockout and rescue cells.

**
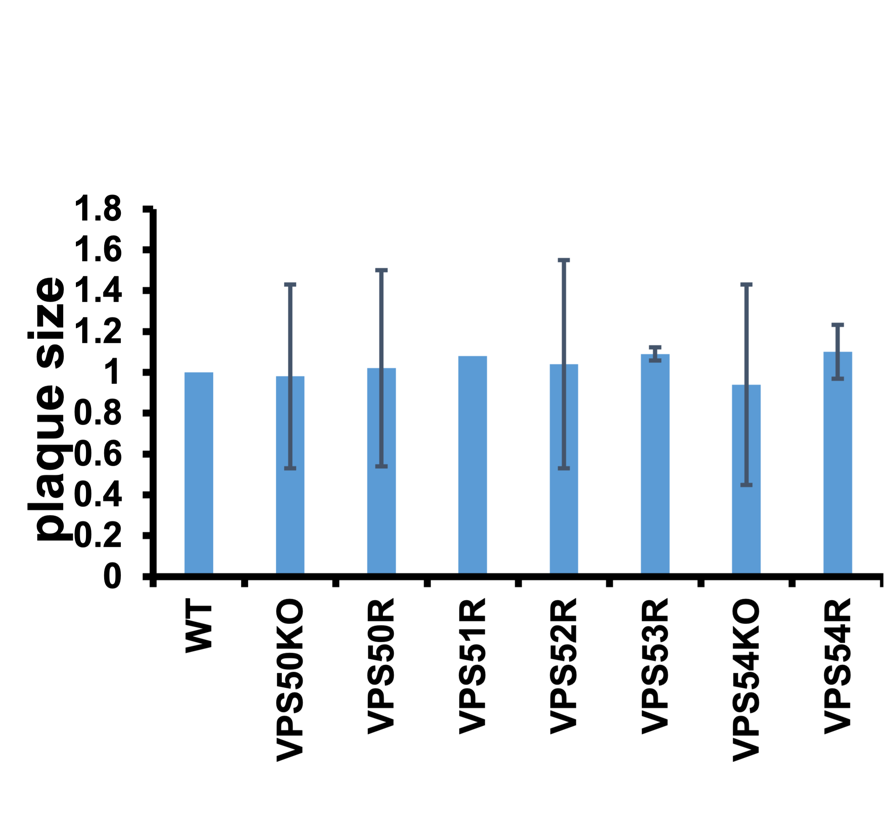
**

**Fig Q. Sizes of BoHV-1 plaques grown in various clones.** ImageJ was using to measure sizes of plaques grown in VPS50KO, VPS51KO, VPS52KO, VPS53KO and VPS54KO (denoted as -/-) cells as well as KO clones with overexpression of corresponding subunit (pVPS50,51,52,53,54). Cas9+/+ clone P was used as control and plaque size for the control was normalized to 1 (n.s.: p>0.05 based on ANOVA followed by t-tests between genotypes).

# Fig R. Plaque size in VPS50KO and VPS54KO cells.

**
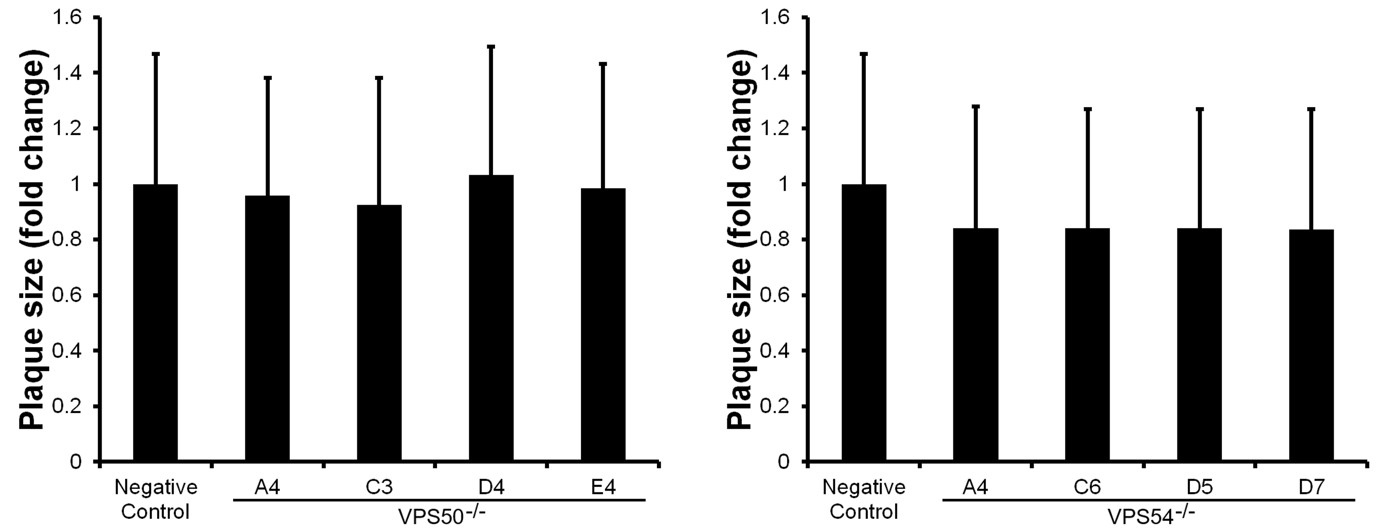
**

**Fig R. Plaque size in VPS50KO and VPS54KO cells.** Plaques were grown and measured in Control cells (Clone P), VPS50KO (left panel) clones or VPS54KO clones (right panel). Average plaque size from Clone P control cells were set as 1 (n.s.: p>0.05 based on ANOVA followed by t-tests between genotypes).

# Fig S. An antibody against VP8 of BoHV-1

#### An antibody against BoHV-1 strain "Schönböken" was obtained from the Friedrich-Loeffler-Institut (FLI) (<https://www.european-virus-archive.com/antibody-or-hybridoma/murine-mab-anti-envelope-protein-bovine-herpesvirus-1-bohv-1>). The antigen used to generate the antibody was described as purified envelope protein of BoHV-1 (as on the above webpage and by personal communication with Dr. Sven Reich at FLI). However, due to the loss of records through time, the exact identify of the antigen became unknown. To test its specificity by western blotting, the antibody was used to stain lysates of concentrated viruses from tissue culture media of infected cells along with an anti-GFP antibody (for detecting VP26-GFP). In addition to a band matching the size of the VP26-GFP (in yellow, ~41kD), we observed one other predominant band around 100kD. This was assigned as the targeted envelope protein by the FLI antibody (Fig SA).

#### To reveal its identity, these samples were then stained with Coomassie blue after resolution by SDS PAGE (Fig SB). Gel pieces spanning around ~100kD were extracted and protein contents within these gel samples were examined by Mass Spectrometry (Fig SC). We found that the most abundant envelope proteins within these samples were gB(103kD), gE(63kD), gM(44kD), gG(49kD) and gH(92.5kD). Unfortunately, we also detected low quantities (less than 5% of the abundance in WT samples) of some of these proteins in our MOCK samples, possibly due to cross-contamination during sample loading and gel extraction. Nevertheless, when we over-expressed these candidate envelope proteins from gB to gH individually, we failed to detect any reactivity between the FLI antibody and these glycoproteins (Fig SD, bottom panel), even though the proteins themselves were properly expressed judging by the western blotting against the C-terminal FLAG tags (Fig SD, top panel). These results suggest that the FLI antibody does not target any of these envelope proteins we suspected.

#### We then focused our efforts on the other proteins detected by the Mass Spec. We found that the tegument protein VP8, being the most abundant viral protein detected, has a molecular weight (93kD) that matches well with the major bands detected by western blot and Coomassie stain (Fig SA and SB). We then proceeded with testing VP8 by cloning, overexpressing and western blot just like before and found that the FLI antibody labelled the VP8 protein with high specificity. In conclusion, our experiments show that the antigen target of this FLI antibody is the VP8 tegument protein of BoHV-1 and the antibody has high specificity against this target.

**
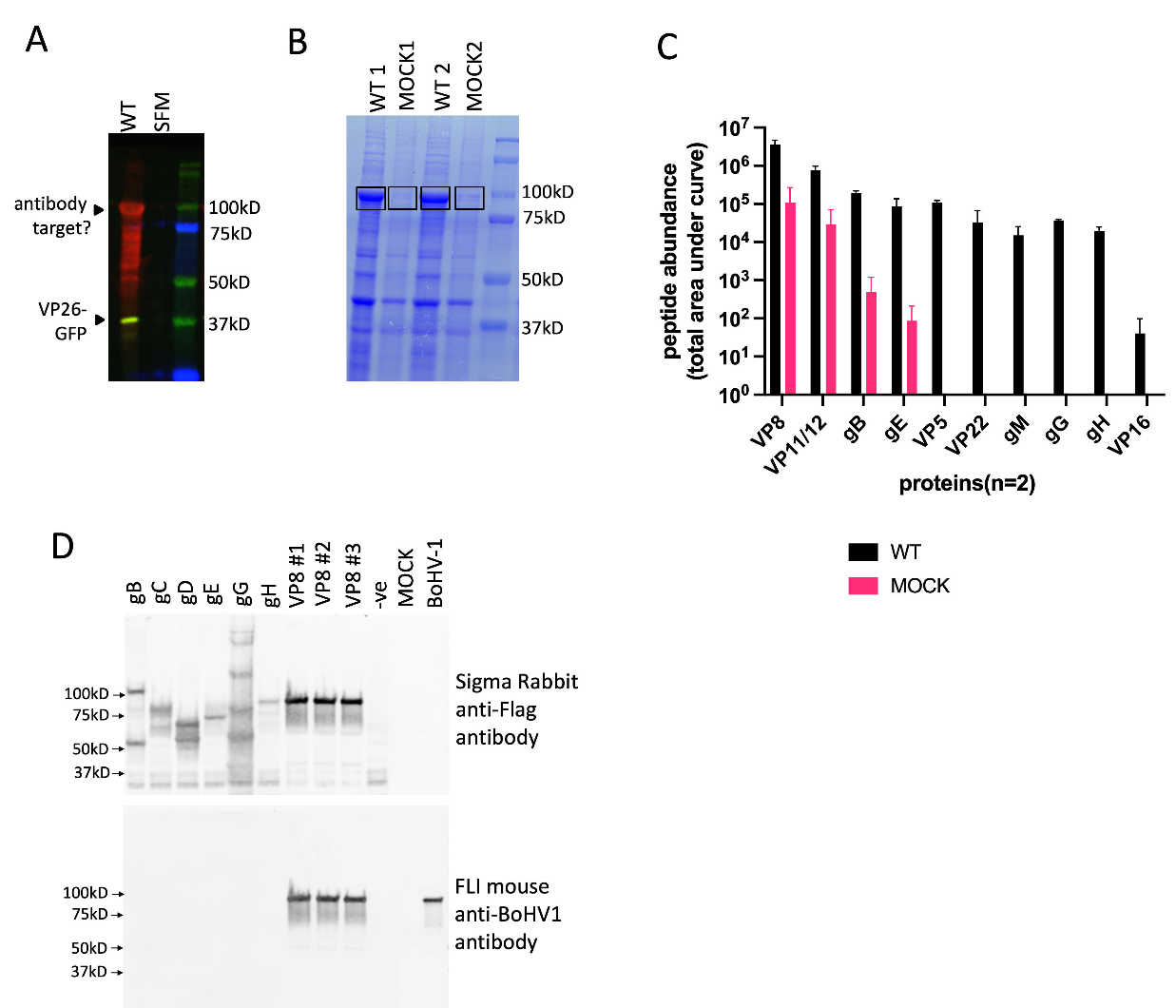
**

**Fig S. Specificity of an antibody against VP8 of BoHV-1.** (a). Western blot result using the anti-BoHV-1 antibody (red) and an anti-GFP antibody (yellow) against a concentrated virus sample harvested at 48h.p.i. (MOI=3) from tissue culture supernatant of WT cells grown in serum free media (SFM, DMEM+1% Pen/Strep). Black arrows point to bands that match the size of VP26-GFP (41kD) and the most prominent band on the membrane, marked with a “?” as the candidate target antigen for the FLI antibody. SFM was used as a negative control. (b). Concentrated virus samples resolved on 10% SDS PAGE gels and stained by Coomassie. PAGE gel pieces (marked by the black rectangular boxes) containing bands that match the size of the detected candidate band by western blot in A were cut out and protein contents were examined by Mass Spectrometry. (c). Proteins with the most abundant peptides in viruses harvested from the WT cells and the MOCK samples detected by the Mass Spectrometry (n=2). The Y-axis represents abundance of each protein as total area under curves of all peptides returned for that protein. (d). Representative Western blot results on lysates harvested from HEK293FT transfected with plasmids overexpressing glycoproteins or VP8 of BoHV-1. The same samples were hybridized with the primary anti-Flag antibody (rabbit) and the FLI antibody (mouse) together followed by secondary antibodies labelling the anti-Flag (Licor Donkey anti-rabbit 800) or the FLI antibody (Licor Donkey anti-mouse 680). -ve: empty vector, MOCK: lysate from supernatant of MOCK infected cells; BoHV-1: lysate from concentrated supernatant of cells infected with the GFP tagged BoHV-1.

# Table A. Numbers of genes targeted, and guides included in the btCRISPRko.v1 library

| **Total genes targeted** | 21,165 |
| --- | --- |
| **Total targeting guides** | 94,000 |
| **No. genes with 6 guides** | 2 |
| **No. genes with 5 guides** | 9,704 |
| **No. genes with 4 guides** | 11,238 |
| **No. genes with 3 guides** | 112 |
| **No. genes with 2 guides** | 71 |
| **No. genes with 1 guide** | 38 |
| **Control guides** | 2,000 |
| **Total guides** | 96,000 |

# Table B. Libraries produced from this study

| **library** | | **Delivery**  **method** | **scaffold** | **colony-based QC** | | **Sanger QC**  **% correct** | **NextSeq-based QC** | |
| --- | --- | --- | --- | --- | --- | --- | --- | --- |
|  |  |  |  | **depth** | **background** |  | **coverage** | **% correct** |
| K2g2 | lentivirus | | Zhang(6) | 1361x | 0.6% | 21/22(95.5%) | > 99.70% | N.D. |
| K2g5 | lentivirus | | Chen(1) | 1300x | 1.5% | 22/23(95.7%) | > 99.9% | 96.02% |
| PBg2 | piggyBac | | Zhang | 4700x | 0.2% | 24/24(100%) | N.D. | N.D. |
| PBg5 | piggyBac | | Chen | 1270x | 0.3% | 21/23(91.3%) | N.D. | N.D. |

**Notes:** K2g2: lentivirus library with the original sgRNA scaffold; K2g5: lentivirus library with the optimized sgRNA scaffold; PBg2: PiggyBac library with the original sgRNA scaffold; PBg5: PiggyBac library with the optimized scaffold; N.D.: not determined.

# Table C. Number of cells recovered from 1^st^ screen

|  | **GFP Negative** | **GFP Low** | **GFP Medium** | **GFP High** | **Non-infected** |
| --- | --- | --- | --- | --- | --- |
| **Repeat 1** | **1.07** | **3.88** | **5.47** | **11** | **50** |
| **Repeat 2** | **1.2** | **3.84** | **6** | **15.7** | **50** |
| **Repeat 3** | **0.53** | **1.11** | **2.59** | **6.76** | **50** |
| **Repeat 4** | **1.07** | **3.6** | **7.01** | **23** | **50** |

**Note: Cell numbers are in Millions.**

# Table D. Number of cells recovered from 2^nd^ screen

|  | **GFP Negative** | **GFP Low** | **GFP Medium** | **GFP High** | **Non-infected** |
| --- | --- | --- | --- | --- | --- |
| **Repeat 1** | **1.66** | **3.1** | **2.53** | **2.45** | **70** |
| **Repeat 2** | **4.3** | **4.3** | **3.5** | **3.3** | **70** |
| **Repeat 3** | **3.6** | **5.4** | **4.9** | **3.7** | **70** |
| **Repeat 4** | **2.14** | **3.0** | **3.27** | **4.66** | **70** |

**Note: Cell numbers are in Millions.**

# Table E. Knockout clone genotypes

| **Purpose** | **Clone I.D.** | **Genotype** |
| --- | --- | --- |
| TRIM5a^-/-^; Cas9^+/+^ | 1B2 | -13/-13 |
| TRIM5a^-/-^; Cas9^+/+^ | 1C1 | +2/+2 |
| TRIM5a^-/-^; Cas9^+/+^ | 2A5 | -2/-10 |
| TRIM5-/- | A44 | -1/-1 |
| TRIM5-/- | B13 | -29/-35 |
| VPS52KO | B1 | -8/-11 |
| VPS52KO | B4 | -5/-10 |
| VPS52KO | C1 | +8/+8 |
| VPS52KO | C4 | -8/-8 |
| VPS51KO | A9 | -1/-2 |
| VPS51KO | B7 | -5/-5 |
| VPS51KO | C2 | -2/+1 |
| VPS51KO | C3 | +1/+1 |
| VPS53KO | E2 | -5/-7 |
| VPS53KO | E5 | -5/-7 |
| VPS53KO | F11 | -2/-2 |
| VPS53KO | F3 | -2/-17 |
| VPS54KO | A4 | +1/-7 |
| VPS54KO | C6 | -5/-5 |
| VPS54KO | D5 | -5/-10 |
| VPS54KO | D7 | +1/+1 |
| VPS50KO | C3 | -1/+25 |
| VPS50KO | A4 | -950/-950 |
| VPS50KO | D4 | -950/-950 |
| VPS50KO | E4 | -950/-950 |
| VPS50;54dKO | C3A2 | -1/+25;-2/-2 |
| VPS50;54dKO | D4B4 | -950/-950;-7/-7 |
| VPS50;54dKO | D4F3 | -950/-950;-7/-8 |
| huVPS50-/- | A1 | +1/+1 |
| huVPS50-/- | G2 | +1/+1 |
| huVPS50-/- | B2 | -2/-2 |
| huVPS50-/- | G1 | +1/+1 |
| huVPS51-/+ | H3 | -3/-1 |
| huVPS52-/+ | B8 | -7/wt |
| huVPS52-/+ | D11 | -6/-2 |
| huVPS52-/+ | F9 | -4/wt |
| huVP52-/+ | G10 | +1/wt |
| huVPS54-/- | D6 | -185/-185 |

# References

1. Chen B, Gilbert LA, Cimini BA, Schnitzbauer J, Zhang W, Li GW, et al. Dynamic imaging of genomic loci in living human cells by an optimized CRISPR/Cas system. Cell. 2013 Dec 19;155(7):1479–91.

2. Dang Y, Jia G, Choi J, Ma H, Anaya E, Ye C, et al. Optimizing sgRNA structure to improve CRISPR-Cas9 knockout efficiency. Genome Biology. 2015 Dec 15;16(1):280.

3. Tzelepis K, Koike-Yusa H, De Braekeleer E, Li Y, Metzakopian E, Dovey OM, et al. A CRISPR Dropout Screen Identifies Genetic Vulnerabilities and Therapeutic Targets in Acute Myeloid Leukemia. Cell Reports. 2016 Oct 18;17(4):1193–205.

4. Hart T, Tong AHY, Chan K, Van Leeuwen J, Seetharaman A, Aregger M, et al. Evaluation and Design of Genome-Wide CRISPR/SpCas9 Knockout Screens. G3 (Bethesda, Md). 2017 Aug 7;7(8):2719–27.

5. Yusa K, Zhou L, Li MA, Bradley A, Craig NL. A hyperactive piggyBac transposase for mammalian applications. Proceedings of the National Academy of Sciences of the United States of America. 2011 Jan 25;108(4):1531–6.

6. Cong L, Ran FA, Cox D, Lin S, Barretto R, Habib N, et al. Multiplex genome engineering using CRISPR/Cas systems. Science (New York, NY). 2013 Feb 15;339(6121):819–23.

1. ^†^ Deceased [↑](#footnote-ref-1)
