# Supplementary material for: GARP and EARP are required for efficient BoHV-1 replication as identified by a genome wide CRISPR knockout screen
Source: PLoS Pathog. 2023 Dec 6;19(12):e1011822. doi: 10.1371/journal.ppat.1011822 (PMC10727446; doi:10.1371/journal.ppat.1011822)
Supplement: Attachment — Submitted filename: response_to_reviewers.docx [file ppat.1011822.s010.docx]

Dear Editors and Reviewers:

Thank you very much for taking the time in reviewing our manuscript and providing us with useful comments and constructive suggestions to further improve our manuscript.

Please see below our detailed responses to your suggestions and additional data we have since generated to answer the key questions raised by you.

As requested, we have conducted additional experiments that further prove our hypothesis that GARP and EARP serve as key links for viral protein trafficking, and that the recycling endosomes and the Trans Golgi Network (TGN) can be major sources of membrane vesicles for the envelopment of alpha- and gamma-herpesviruses. The new data produced from these experiments have been summarized below:

We conducted a new 36-hour virus infection time course that measured titers, genome copy numbers and genome to pfu of viruses collected from the supernatants and the cell pellets of VPS52KO cells (deficient in both GARP and EARP) infected at MOI=3. These data are presented in **Fig. S19** and show that viruses released by these deficient cells had a high genome/pfu ratio compared to control cells, which is in agreement with our data collected from cells infected at MOI=0.1 (**Fig. 7B,C,D**) and provide further evidence that the packaging of these viruses in the VPS52KO cells was affected.

We then quantified the gB protein on concentrated cell free viruses produced by the VPS52KO cells and found that viruses released by these KO cells had substantially less gB compared to viruses produced by non-edited cells. We also examined the VPS52KO cells infected by BoHV-1 via confocal microscopy and western blotting but found no substantial reduction in the protein level of late viral genes, including VP26-GFP and glycoprotein B. Based on these data, we conclude that the reduced gB content on viruses produced by the VPS52KO cells was not caused by altered gB protein expression, as mainly suggested by Reviewer #3, but rather the disrupted roles GARP and EARP play during protein and membrane trafficking. All these data summarized here are presented in a new **Fig. 8** on pages 41-42 of our revised manuscript with tracked changes, accompanied by text detailing the data from line 577 to 643.

For minor changes and suggestions raised by the reviewers, please see the point-to-point responses detailed below in this document and changes in the tracked versions of the main manuscript, the “supplementary data” file and the “supplementary materials and methods” file.

To the best of our knowledge, we have addressed or responded to every point or issue raised by the reviewers. Whilst we strived to address every concern as best as we could, due to the lack of resources in livestock research, such as antibodies against other viral and host proteins, we have not been able to perform additional experiments that look at the expression levels of other late viral proteins or their localization at various timepoints of the virus life cycle.

We thank you in advance for your review of our revised manuscript and sincerely hope the new data we have produced and revisions we have made to our manuscript are sufficient for its acceptance by your journal.

Yours,

Spring Tan

**Part I - Summary**
Please use this section to discuss strengths/weaknesses of study, novelty/significance, general execution and scholarship.

- Reviewer #1: For this study, the author utilized a genome wide CRISPR knockout strategy to target all protein coding genes in the bovine genome. As expected, this strategy identified numerous genes that impaired or stimulated viral replication. Interestingly, the Golgi-associated retrograde protein (GARP) complex and endosome associated recycling protein (EARP) complex scored as being very important for virus production. BoHV-1, like most herpesviruses undergoes a complicated strategy to generate infectious viruses that are shed or spread via cell to cell. Hence, it is expected these pathways would be important. With that said, there are several issues that need to be addressed.

***Response:*** *We thank the reviewer for the time spent on reviewing our manuscript. We have attempted to address the issues raised by you below.*

- Reviewer #2: This manuscript by Tan et al. illustrates the careful construction and impressive documentation of a CRISPR/Cas9 knockout library for high throughput genomics and gene discovery in the cattle genome. The btCRISPR library generated, targeting all protein coding genes in the cattle genome, will be hugely useful in studying cellular processes in bovine cells, as well as processes that intersect with viruses infect bovine cells. At every barrier, the authors went the extra mile to create a high-standard resource. The authors perform extensive gene ontogeny analysis, and illustrate the possible involvement of many pro- and anti-viral host genes involved in the viral replication cycle. They validate one of these sets of genes, comprised of VPS 50-54, which assemble to form the GARP and EARP complexes. They then rigorously knock out the components of these complexes to ascertain their role in the BHV1 life cycle. They arrive at an intriguing model that trafficking of viral glycoproteins to RE and the TGN is cut off by GARP and EARP KOs, and the vesicles that bud off are deficient in viral glycoproteins essential for 2ndary envelopment of capsids. This hypothesis is intriguing and prompts some testable hypotheses.

***Response:*** *We thank the reviewer for the critical review of our manuscript. To test the hypothesis proposed in the 1st version of our manuscript which is highlighted by your comments above, we have conducted some of the additional experiments suggested and presented the new data as a stand-alone new figure, i.e.* ***Fig.8*** *on page 41-42 in the main manuscript. The text that describes this figure starts from line 577 and ends in line 643 in the tracked manuscript. Most importantly, by western blotting, we showed that viruses released by VPS52KO cells (missing the GARP and EARP) have a lower glycoprotein B content compared to unedited cells, which is unlikely due to impairment to gB transcription or translation, as we have not observed any of such changes in our experiments (****Fig.7A*** *on page 40 and* ***Fig. 8F,G,H*** *on page 41). These additional data offer strong proof for the involvement of the tethering complexes during secondary envelopment of herpesviruses.*

- Reviewer #3: Tan et al., develop tools for and then perform a screen for host cell gene products that affect expression of one of the BHV-1 viral capsid proteins, VP26, during a single cycle of infection (i.e., after a relatively high m.o.i. infection at times prior to completion of a first cycle of infection). They identify many genes with both positive or negative effects on VP26 expression and show nicely that knock-down or CRISPRi inhibition of expression putative proviral host factors affects initiation of plaque formation by BHV-1. This appears to have been a fruitful screen and the cell line and library tools that they have constructed represent a highly useful and significant tool for identifying pro- and anti-viral factors for BHV-1 expression.

***Response****: We thank the reviewer for the positive feedback on our screens.*

- The paper then takes a confusing turn as the authors focus on two membrane trafficking-related protein complexes, EARP and GARP and attempt to build a case that these are likely to be important for virus assembly. This is confusing in part because their screen was not structured to find cellular factors that promote events in virus replication past the expression of VP26. Indeed, it is difficult to see how factors that might even be absolutely essential for assembly would have had any effect on the outcome of their screen. Perhaps unsurprisingly, the evidence presented that these factors directly affect assembly is incomplete and not easily interpretable.

***Response:*** *Since we didn’t have a functional antibody against a BoHV-1 protein at the time when we were developing the screens, we resorted on the tagged virus with the VP26-GFP expression level as our phenotypic read out. Based on our screens, four out of the five subunits of GARP and EARP were identified, namely VPS51,52,53, and 54 (****Datafiles 2,3,5*** *and* ***Fig. 4E*** *on page 36). To understand any potential roles these complexes could play during BoHV-1 replication, we searched through literature and thought of what we thought was the most likely hypothesis and explanations for our data, that is these tethering complexes are involved in trafficking of viral proteins and cytoplasmic envelopment of BoHV-1. Indeed, it would not have been the most straightforward conclusions to make if we were to solely base our conclusions on the screen data. However, our new data, that viruses released by the knockout cells have significantly less gB on their envelope, offer further support to the conclusions and projections we have mentioned above and in the paper.*

*It is entirely possible that the reduced VP26-GFP protein expression levels in the GARP/EARP knockout(KO) cells, as observed in our validation experiments* ***(Fig. 6D*** *on page 39 and* ***Fig. 8H*** *on page 41), was indeed what enabled us to pick out the tethering subunits from the screens in the first place, since our screen set up was to enrich for cells with Low or High levels of GFP expression. But we think what we have since observed during the validation of these candidates, i.e. disrupted trafficking of glycoproteins and envelopment of newly synthesized capsids, also happened in the cells with GARP/EARP KO during our pooled screen. However, this phenomenon was not captured by our screen simply because that’s not what our screen was designed to measure. Rather, our screen was set up to FACS sort cells based on GFP protein levels in a high throughput and cost-effective manner. If we were able to use titers or the genome/pfu ratio of viruses produced by the pooled cells as our phenotypic readout in a new screen, it is very possible that these GARP/EARP candidates would stand out again. But such a screen requires us to conduct plaque assays or western blots on a single cell level, cheaply and efficiently. It is very desirable for high through functional screens like ours, but unfortunately, such technologies are not here or affordable for genome wide screens yet.*

*Also, upon an extended literature search during our revision, we found two publications that also describe results on the involvement of the GARP during cytoplasmic envelopment of poxviruses. Please follow these links for these papers:*

[*https://journals.asm.org/doi/10.1128/JVI.00011-17?url_ver=Z39.88-2003&rfr_id=ori:rid:crossref.org&rfr_dat=cr_pub%20%200pubmed*](https://journals.asm.org/doi/10.1128/JVI.00011-17?url_ver=Z39.88-2003&rfr_id=ori:rid:crossref.org&rfr_dat=cr_pub%20%200pubmed)

[*https://journals.asm.org/doi/full/10.1128/jvi.01464-16?rfr_dat=cr_pub++0pubmed&url_ver=Z39.88-2003&rfr_id=ori%3Arid%3Acrossref.org*](https://journals.asm.org/doi/full/10.1128/jvi.01464-16?rfr_dat=cr_pub++0pubmed&url_ver=Z39.88-2003&rfr_id=ori%3Arid%3Acrossref.org)

- The authors show that individual knock-outs of three of three subunits common to the EARP and GARP complexes result in strong inhibition of the ability of BHV to initiate plaque formation and have a small effect on the sizes of the plaques that do form (although this latter measurement is not controlled by rescue of expression). They also attempt to show that these knockouts affect virus production in Figure 6D. The problem here is that the experiments shown in Figure 6D do not measure virus production. Rather, they show kinetics of VP26 expression. The lower graph is particularly telling in that it measures VP26 expression in a single cycle of infection (i.e., the vast majority of cells infected at m.o.i. = 3). This result strongly suggests, despite what is shown in Figure 7, that the primary defect in their VPS51/52/53 knockouts is a strong inhibition of expression of at least some late genes. This is, of course, consistent with the results of the screen in which these candidates were identified. The authors show in Figure 7 that the virions produced in the VPS52 knockout have a lower specific infectivity than those from a wild-type cell clone. This can easily be explained as an indirect effect of inhibition of expression of critical virion components. It should also be noted that the experiments shown in Figure 7 are also not controlled with cells in which the knockout is repaired.

***Response:*** *The reviewer is correct in pointing out that* ***Fig. 6D*** *measures the dynamics of VP26-GFP protein expression, not the actual viruses produced. However, we disagree that the reduced virus titer and genome/pfu ratio of viruses produced by these KO cells can be simply explained by altered viral protein expression, such as that of VP26-GFP. For the time course conducted with MOI=3 (bottom panel Fig. 6D on page 39), a 2.9-fold reduction in GFP intensity was observed at 24hpi, which is a lot less severe than the 2-3 log reduction in virus titer (****Fig. S19A*** *and* ***Fig. 7B****). We don’t think the reduction in GFP could fully explain the loss of virus titer. In addition to VP26-GFP, we also investigated gB, which is another late gene, and found that there was no significant reduction in gB at 10 h.p.i by confocal microscopy (****Fig. 8A****) or at 48 h.p.i by western blotting (****Fig. 8F,G****). Instead, we observed a 10-fold decrease in gB content on cell free viruses released by GARP/EARP null VPS52KO cells. In combination with the high genome to pfu data* ***(Fig. 7D****), we think the impairment in infectious virus production by these KO cells could be a combinatorial effect from inhibited protein expression of viral proteins we have tested (VP26-GFP) or those yet to be tested, and disrupted envelopment and packaging due to the loss of GARP/EARP, with the latter being the most responsible, as supported by our strong genome/pfu data and reduced gB content on cell free viruses produced by the KO cells.*

*As to the experiments in Fig. 7 not controlled with the rescued cells, since we have already shown that overexpressing the missing subunits in the KO cells completely restored the impairments in virus production (****Fig. 6B,C,D,E****), we decided not to include the rescue cells in our follow-up experiments, since we already had large numbers of samples to process and measure for the time-course experiments.*

- The approaches shown in this manuscript for library generation screening and validation are admirably state-of-the-art. However, to draw the conclusion that the EARP and GARP complexes are directly important in virus assembly, the authors will need to incorporate additional approaches. They must, first, assess the expression of viral proteins (in addition to mRNA) to determine the effects of their knockouts on expression. They must also perform actual virus production assays (i.e., single- and multi-step growth experiments that measure production of infectious virus rather than marker gene expression). Also, there is no TEM or imaging of any kind to support an effect on virus assembly.

***Response:*** *We thank the reviewer for the positive feedback on the screens we produced. Regarding any potential changes on the viral protein levels, we have since examined that of glycoprotein B and VP26-GFP in the VPS52KO cells (deficient in both GARP and EARP) by confocal microscopy (10 h.p.i. at MOI=1,* ***Fig. 8A****) and western blotting (48 h.p.i at MOI=3,* ***Fig.8F,G,H,I****). In all cases, we did not observe a dramatic decrease of viral protein expression. Instead of reduced gB levels, we observed a 2-fold increase of gB/GFP ratio in the KO cells compared to the controls (****Fig.*** ***8F,I****). Please see new text from 448-489 in the manuscript and line from the revised “supplementary materials and methods” for a more detailed description of these experiments and results. For the virus growth curves, we included a growth curve on virus titers measured by plaque assays for the 52KO cells and control cells infected at MOI=0.1(please see* ***Fig. 7B****) in our original submission. We apologize this information was not straightforward to find. We have since edited the legend to help with this (see line 1130 - 1131). Since then, we have also produced a new series of virus growth curves from cells infected at MOI=3 (see* ***Fig.*** ***S19*** *on page 16 in the revised supplementary data file).*

- More minor points include:
  Figure 1B assay is not explained. This is presumably output from a T7E1 assay, but the basis of the assay as applied in this circumstance is unexplained. Insufficient information is provided to allow critical evaluation of the data. And this is a problem throughout the portion of the paper that describes cell line and library engineering.

***Response:*** *We have now added text describing the T7E1 assay from line 166 to 184 and in the legend of* ***Fig. 1B*** *from line 1075 to line 1077 to help explain the assay, and how it was used to evaluate the efficiency of CRISPRs and TALENs produced to target TRIM5a. We have also made minor edits throughout the manuscript to include more details that we think might help with readability.*

- Also, no validation of knock-out of TRIM5alpha expression is presented. The authors get the effect they want with some clones, but whether this is related to TRIM5 KO is not clear. In one respect, it does not matter, since they get clones that show elevated transduction efficiency, but the lack of transparency and rigor in characterization of those clones is disturbing.

***Response:*** *Due to the lack of a functional antibody for bovine TRIM5, we were unable to show the KO on the protein level. However, all these clones that exhibited elevated transduction efficiency were genotyped by sanger sequencing and contained frame shifting indels that lead to early truncation of the TRIM5 protein based on in silico prediction.*

- Given that the screen was based on diminished or enhanced GFP expression in a single cycle of virus replication, how do the authors imagine, they identified genes that are involved in virus assembly and virion specific infectivity?

***Response:*** *Please see response made previously from line 58 to line 89 in this document.*

- Table S1 – the meaning of the numbers in the right-hand column is not explained. The table is unintelligible.

***Response:*** *We apologize for the confusion. We have since re-formatted the table that show the numbers of genes targeted and guides included in the library (line 776-779 in the revised and tracked “supplementary data” document).*

- Figures S3-S5 do not measure the parameter relevant to their screen. They look, on the one hand, at ability to initiate a plaque and, on the other, at the ability to spread. This is a problem because their screen is based on inhibition or enhancement of late protein expression during a single cycle of infection. The relationship between plaque formation, spread and virus single-cycle replication is complicated (at least in the alphaherpesviruses) and the authors should validate the utility of their cell lines using the same sort of criteria they will use for their library screen.

***Response:*** *The main purpose of these experiments was to decide whether Cas9 overexpression and TRIM5-/- would affect infectivity by the BoHV-1 virus so that we choose/engineer a cell line suitable for the screen that still behaves like the wt cells, and that’s what we have learned from experiments presented in Fig. S3-S5. And based on these data, we decided to use the Cas9+/+;Trim5-/- cells for our screens. To reduce the chance of false positives due to Cas9 overexpression, TRIM5 KO or other factors, we conducted our genome scale KO screens in the TRIM5-/-; Cas9+/+ cells, with 2,000 non-targeting sgRNAs as internal negative controls. During validation we generated all KO clones for the candidate genes based on the Cas9+/+ clone with functional TRIM5 (Clone P). In addition, we used Clone P and pure WT cells as controls in all our key validation experiments.*

- Figure 2C axes are uninterpretable. Y axis: “Density” of what? What are the units? X axis: is this nucleotides?

***Response:*** *We have rewritten the legend for* ***Fig. 2C*** *from line 1109-1110.* *This is a probability density plot to show the frequency of guides that cut within a certain range of positions relative to the common coding sequence for every gene targeted. For example, the plot shows that 93.3% of all the guides in the library cut within 0% to 50% of the full-length peptides of the targeted genes.*

**Part II – Major Issues: Key Experiments Required for Acceptance**
Please use this section to detail the key new experiments or modifications of existing experiments that should be absolutely required to validate study conclusions.

Generally, there should be no more than 3 such required experiments or major modifications for a "Major Revision" recommendation. If more than 3 experiments are necessary to validate the study conclusions, then you are encouraged to recommend "Reject".

- Reviewer #1: 1. This manuscript contains 16 supplementary Figures. The authors need to include tables or a couple of figures that summarizes what the top 10 genes or signalling pathways that were identified and include it in the manuscript. Furthermore, these genes need to be named and not just listed as number, and a brief description included.

***Response:*** *We have added tables of contents to the “supplementary data file” and to the “supplementary materials and methods file” to help with navigating the data. We have also prepared a table that lists the top 10 pro- and anti-viral genes validated by the focused screen. The table is presented as the new* ***Fig. 4G****.*

- 2. What was the rational for examining Circ RNA expression? It would have been much better to look at ICP0 or ICP4 expression was because it is known these viral genes are crucial viral regulatory genes.

***Response:*** *We have since profiled the expression of ICP0 and included the data as a plot in* ***Fig. 7A****. Like all the other three genes we tested, we observed no difference in ICP0 transcription efficiency between non-edited cells and VPS52 KO cells upon BoHV-1 infection.*

- Reviewer #2: The manuscript is so thorough in describing the construction of the library, but the validation experiment and the single experiment to ascertain the roles of GARP/EARP in viral life cycle are a bit lacking. Figure 7 demonstrates that the genomes/pfu ratio is much higher in cells that lack the interchangeable GARP/EARP complex members, suggesting the model proposed in Figure 8: that trafficking of viral glycoproteins to RE and the TGN is cut off by GARP and EARP KOs, and the vesicles that bud off are deficient in viral glycoproteins essential for 2ndary envelopment of capsids. But Figure 7 - is where the investigation of GARP/EARP begins and ends.

  1. Given that these VPS proteins are involved membrane trafficking, it is unclear to what extent virions are produced.
  For example, do BHV virions lack gB or other glycoproteins?
  BHV viral particles should be purifiable. And also should be GFP+.
  Particle:pfu ratios using electron microscopy to show that properly or improperly-formed virions are being made.
  Better still, if an Ab exists, immunoelectron microscopy could be performed to show that viral glycoproteins are reduced in the viral envelope.
  Are there are normal number of virions in GARP/EARP KO cells? Is their morphology affected?
  Could the authors examine membrane protein content of virions from cells deficient in EARP/GARP? Or examine expression of gB and localization of gB at various time points during wt infection of control cells vs. EARP/GARP deficient cells.

***Response:*** *We have taken the opportunity to conduct additional experiments utilising an antibody that is likely to be specific for the gB of our virus (please see our western and mass spec data that demonstrates this as presented in* ***Fig. S20*** *on page 17 of our revised and tracked “supplementary data” file). By ultra-centrifugation of viruses in tissue culture supernatants and western blotting, we found that the gB content on viruses released by the VPS52KO is dramatically reduced to ~10% of the viruses released by the control cells (****Fig. 8B,C,D*** *on page 41 and associated text from line 573 to 643). By confocal microscopy and western blotting, we showed that this reduction is not due to altered expression level of the gB protein in the VPS52KO cells, on the contrary, we observed elevated levels (~2 folds, relative to the level of VP26-GFP) in the VPS52KO cells compared to the controls (****Fig. 8A,F,I****). Due to the lack of antibodies that work well in cows, we have not been able to investigate the localization of gB in detail. Out of all the antibodies we have tested, only one worked in our hands that targets protein gm-130 which is a cis Golgi marker. And we have not observed any obvious difference in the distribution of gB relative to gm-130 in the KO cells compared to the wt and Cas9+ cells (****Fig. 8A****).*

- 2. Lines 363- Strange that HSV-1 results in a relatively tiny (but reproducible) reduction in titer – the fold reduction is not mentioned (and should be) but it looks like a 2-4 fold reduction? This is strangely minimal compared to the 3 log reduction in AIHV titres, a gamma herpesvirus. These seem to be hugely different and at a minimum, worthy of some comment in the discussion. The last sentence says “indicating that AIHV and HSV might employ GARP and EARP similarly to BHV”. If GARP/EARP affect BHV and AIHV titres by 3 logs and HSV titres by 3-fold, are they really functioning similarly?

***Response:*** *Thank you for pointing this out. The modest reduction of HSV1 titer could be because we were unable to generate bi-allelic KO clones for VPS51, VPS52 and VPS54 in the human carcinoma cell line A549 (****Fig. 6F****), so mono-allelic knockout cells without complete disruption of the complexes* *were used for the infections, whereas for the BoHV-1 experiments bi-allelic knockout cells were used. More work is needed to confirm the negative impact the loss of GARP/EARP on HSV-1 replication. And we have now highlighted on this in the text (see lines from 508-509).*

- Line 369 – In fig F, for HSV1, VPS54 -/+ results in the largest reduction in titres at ?? 5X, compared with ? 2.5X reduction with VPS50-52. Yet for AIHV and BHV, the VPS54KO does almost nothing to the AIHV titers (fig H), and the VPS 51-52 reduce titers by 3 logs. This seems to argue that HSV is different from AIHV and BHV. Some discussion is warranted, if not further experimentation.

***Response:*** *Please refer to the response made above in lines 270-275.*

- 3. GARP and EARP KO affected viral entry and did not affect viral gene transcription, as ascertained through examination of vDNAout and transcription of IE, E, and L genes.
  If transcription of gB is unaffected, and if GARP/EARP affect virion integrity by altering trafficking of gB, then perhaps gB is mislocalized within infected cells?

***Response:*** *Our new data shows that viruses produced by the GARP/EARP KO cells have reduced gB level, likely due to disruption to its trafficking and possible mis-localization. Due to the lack of antibodies that label cytoplasmic organelles other than GM130 however, we were unable to produce direct evidence of this by immunofluorescent staining and confocal microscopy.*

- Reviewer #3: They must, first, assess the expression of viral proteins (in addition to mRNA) to determine the effects of their knock-outs on expression. They must also perform actual virus production assays (i.e., single- and multi-step growth experiments that measure production of infectious virus rather than marker gene expression). Also, there is no TEM or imaging of any kind to support an effect on virus assembly.

***Response:*** *Please see lines 137-149 in this document for the response to a similar suggestion.*

**Part III – Minor Issues: Editorial and Data Presentation Modifications**
Please use this section for editorial suggestions as well as relatively minor modifications of existing data that would enhance clarity.

Reviewer #1: (No Response)

- Reviewer #2: 1. The methods describing the expts in Figure 7 are difficult to find and may not exist. In Figure 7BCD they must have infected either clone P or the 52KO cells with BHV1, then taken the supt, pellet, and total, at different time points, and then titer what is collected at these time points on some other type of cell using the methods described in “Plaque assays”. No mention is made of this expt in the methods or legend.

***Response:*** *We apologise for this oversight and have since included the complete experimental procedures carried out for* ***Fig. 7*** *and* ***Fig. S19*** *in the revised and tracked supplementary materials and methods document (lines 474-500).*

- 2. Do not understand “n.s.” in Figure D. Error bars are minute or nonexistent? At the 36 hr timepoint, the KO is at 10^3 and the wt is at 1. How could this be n.s., while the differences in C look negligible, with overlapping error bars, and yet are marked with 1 to 4 ****? Not clear to which data points the asterisks in Fig 7C refer. A particular time point only? Which one? Why only one? Why are there error bars only on some points but not others? What is the number of biological replicates?

***Response:*** *We apologize for this error partly due to missed information during copying and pasting. We originally plotted the data on a log scale showing mean +/- 1 STD for* ***Figures 7B-D****. However, due to the scaling some error bars appeared to be too small to be visible. We then conducted Two-way ANOVAs followed by multiple comparisons for each of the nine series of data presented, and only reported the single p-value returned by the two-way ANOVA comparisons for the entire time course series. We have since replotted the data series, showing every data point on the plots to better visualize the variability. As to the statistical analysis for every set of data, we conducted student’s t-tests after the two-way ANOVA at each time point and only highlighted those with p values of significance. Due to the high variability of the KO clones and our experimental procedure, even though the differences in averages between 52KO cells and control cells (effect sizes) are big for some timepoints, the statistical analysis did not return significant p-values, they are small (0.1 – 0.2) but not smaller than 0.05.*

- 3. Figure 6 H – cannot find legend for panel H. Must have been intended to be part of panel G. Text references 6H, though.

***Response:*** *We apologize for this error. The labels for the y-axis have since been added to* ***Fig. 6H*** *on page 39.*

- 4. Figure 7 depicts relative genomes, relative mRNA/vDNA, relative vDNA/pfu – relative to what? cannot find or missed description of relativity in legend or Methods.

***Response****: Please see lines 474-487 on page 8 of the supplementary materials and methods document with tracked changes.*

- 5. Line 420 – Combined with existing knowledge … - what knowledge is this? References?

***Response****: We have since added the references to line 692.*

- 6. Line 415 – we conducted a series of experiments to understand the routes these complexes are recruited by the virus for self-benefit. (?? A hybrid of two sentences ?)

***Response:*** *we have since rewritten this in lines 657-659.*

- 7. Line 433-34 referring to colocalization could use some references.

***Response:*** *We have since added the references in line 590.*

- 8. Line 336 – would be clearer if text read “VP26-GFP” synthesis (Fig 6D left panel).

***Response:*** *We have since changed “VP26 synthesis” into “VP26-GFP synthesis” on line 441 in the tracked document.*

- 9. Line 336 - Figure 6D does not have a left panel…Also the abscissa is unlabeled in Fig 6D top panel

***Response:*** *We apologize for this editing error due to rearrangements of figures. “Left” has now changed to “top” and “right” has been changed to “bottom” on line 443.*

- 10. “We also tracked the rate of VP26 synthesis in these KO cells (Fig. 6D left panel) by measuring GFP fluorescent intensity from 0 h.p.i. to 72 h.p.i, following infection with GFP tagged BHV-1 (Fig. 6D right panel)” Line 336 - It seems that the difference between the top and bottom panels are the MOI. Both panels depict the KO cell lines, although only the top has a legend. And both panels look like they are following infection with GFP-BHV? This sentence is confusing, as the distinction between left and right panels does not seem to correspond even to the top and bottom panels in Fig 6D.

***Response:*** *We apologize for the negligence and confusion caused. It’s due to rearranging the panels and forgetting to update the text. The text has now been fixed on line 423 to reflect this.*

- 11. Line 136 and prior – please define btau5.0.1 and rosa26 and TAL1.6.

***Response:*** *btau5.0.1 has now been defined in line 196 and rosa26 has now been defined with reference on line 154. Regarding TAL1.6, we have now defined TAL1.6 from line 153-154.*

Reviewer #3: (No Response)

**Additional comments raised by reviewer:**

- Bovine herpesvirus 1 is now abbreviated as BoHV-1, not BHV-1.

***Response:*** *We have since changed our abbreviation to BoHV-1 according to the reviewer’s suggestion. 73 replacements of “BHV-1” with “BoHV-1” have been made throughout the manuscript.*

- 2. GARP and EARP were not defined in the abstract.

***Response:*** *They are now defined in the abstract from line 39 to line 40 as “endosome-associated recycling protein (EARP) complex and Golgi-associated retrograde protein (GARP) complex”.*

- 3. Is it accurate to state that GARP and EARP are redundant functions that BoHV-1 needs for retrograde transport and virion production?

***Response:*** *We have rephrased “redundant” as “alternative” on lines 495 and 1226 of the tracked manuscript.*

- 4. One major, but unavoidable, flaw with this approach is cellular genes necessary for cell growth will not be viable and thus cannot be tested. This should be discussed in the manuscript.

***Response:*** *We have added discussion on the study of fitness genes using CRISPRko approaches from line 778 to line 781 in the tracked main document.*

- 5. Figure 6F: the differences were less than one log in virus production. Are the authors sure this difference is biologically relevant?

***Response:*** *Please see response to a similar point raised by another review from line 270 to line 275 of this document.*
