# Supplementary material for: GARP and EARP are required for efficient BoHV-1 replication as identified by a genome wide CRISPR knockout screen
Source: PLoS Pathog. 2023 Dec 6;19(12):e1011822. doi: 10.1371/journal.ppat.1011822 (PMC10727446; doi:10.1371/journal.ppat.1011822)
Supplement: Attachment — Submitted filename: response_to_reviewers.docx [file ppat.1011822.s011.docx]

Dear Editor and Reviewers:

Thank you for taking your time to review our revised manuscript.

# The main concern raised by you was about the identity of the BoHV-1 antigen targeted by our antibody. In the previous revision, we thought it to be the glycoprotein B (gB) of BoHV-1. According to the communication and records from the Friedrich-Loeffler-Institut (FLI) received with this antibody, its target was a purified envelope protein of BoHV-1. Since gB was the most abundant envelope protein detected by our Mass Spec experiments with a matching size to the protein that reacted with this antibody based on western blotting, it was the most fitting candidate.

# Fortunately, and thanks to the valuable doubt raised by you, it prompted us to examine the antibody more closely. And by overexpressing all candidate viral proteins individually in cells, we have since realized that the real target of this FLI antibody is indeed not gB, but the tegument protein VP8. Accordingly, the knockout of the tethering complexes GARP and EARP disrupted the packaging of VP8, producing cell free viruses that severely lack VP8 in the tegument. Please see our response below and a revised Figure S19 on pages 16-17 of the tracked “supplementary data” for more information on this.

# Even though the identify of our protein of interest is very different from what was originally thought, this new information does not affect the major conclusions we have made in this manuscript. It did help us refine the viral protein trafficking model we have proposed in the original revision. Based on the new data and refreshed literature search, we now propose that the GARP and EARP are involved in the trafficking of gM, which interacts closely with VP8 and is responsible for the correct localization of VP8 in the cytoplasm and its proper packaging into virions. Please kindly review this updated model, presented as a revised Figure 9 on page 44 in our tracked “main_text” document. In addition, please also review our point-to-point responses to your minor suggestions bellow.

Please also accept our sincere apology for this genuine mistake we have made. The antibody was generated in 2005, it could well be that there was limited knowledge on VP8 back then and it was suspected as an envelope protein hence labelled as such. We remain hugely grateful to the Friedrich-Loeffler-Institut for making this antibody freely available to us. It contributed greatly to our further understanding of tethering complexes GARP and EARP and how they could be involved in the trafficking of viral proteins. To make more people benefit from this antibody, we will communicate our results to the FLI institute so that the information on this antibody can be updated on the records.

We thank you again for your patience and valuable advice that have improved our manuscript substantially.

Sincerely yours,

Spring Tan

Reviewer Comments (if any, and for reference):

Reviewer's Responses to Questions

**Part I - Summary**
Please use this section to discuss strengths/weaknesses of study, novelty/significance, general execution and scholarship.

- Reviewer #2: This manuscript by Tan et al. illustrates the careful construction and impressive documentation of a CRISPR/Cas9 knockout library for high throughput genomics and gene discovery in the cattle genome. The btCRISPR library generated, targeting all protein coding genes in the cattle genome, will be hugely useful in studying cellular processes in bovine cells, as well as processes that intersect with viruses infect bovine cells. At every barrier, the authors went the extra mile to create a high-standard resource. The authors perform extensive gene ontogeny analysis, and illustrate the possible involvement of many pro- and anti-viral host genes involved in the viral replication cycle. They validate one of these sets of genes, comprised of VPS 50-54, which assemble to form the GARP and EARP complexes. They then rigorously knock out the components of these complexes to ascertain their role in the BHV1 life cycle. They arrive at an intriguing model that trafficking of viral glycoproteins to RE and the TGN is cut off by GARP and EARP KOs, and the vesicles that bud off are deficient in viral glycoproteins essential for 2ndary envelopment of capsids, is intriguing and prompts some testable hypotheses. In Figure 8, the authors then test the hypothesis that gB is reduced or absent from released cell-free virions and show that virions generated from VPS52KO possess less gB, supporting the idea that GARP/EARP participate in the normal trafficiking of gB.

**Response**: Thank you very much for the positive review of our manuscript.

- Reviewer #3: The revised manuscript of Tan et al., addresses many of the concerns raised in their first submission. The authors have made an effort to address the possibility that there are real effects on virus production due to GARP/EARP subunit knockout (Figure S19) and that that these effects on virus production are due to changes in protein expression rather than effects on assembly (Figures 8 and S20). They are almost there, but there are problems with each of these three critical figures that should be addressed before publication.

**Response**: Thank you very much for your constructive feedback. Please see below for our point-to-point responses to your review.

- Figure S19 is not easily interpretable. The Panel B Y axis is labeled “vDNA copies (relative).” Relative to what? A ratio relative to a cellular housekeeping gene would make sense for total and pellet values, but not for supernatant where the presence of host DNA would indicate cell lysis. The authors should indicate what is actually being measured in this panel. In panel C, the numbers do not make sense. The Y axis is “vDNA/pfu (relative).” If you take any of the time points in panels A and B and calculate the vDNA/pfu ratio, you do not get a value that corresponds to a point in panel C. To give just one example, the clone P 12 h time point for total production, vDNA (relative)/pfu = ~1e2/~1e6 = 1e-4. The value actually plotted in panel C is ~5e0. The trends look about right, but the authors need to explain how the data was actually derived.

**Response:** We apologize for the confusion we have caused due to our wording. To make it clearer, we have replaced “(relative)” with “(normalized)” in all the panels included in **Fig. 7B,C**. There is also a brief description on how the data were processed prior to the plotting in the tracked “supplementary materials and methods” file, from line 419 to line 432 on page 8. Essentially, within each data series we normalized all calculated data points to the smallest value which was set as 1 and these normalised values not the original calculated values were then used to generate the plots.

- The data in Figure S19 is also critical for the conclusions that suthors wish to draw in the paper and should be in the main body of the paper rather than the supplementary data.

**Response:** Thank you for the suggestion. Figure S19 has now been moved to the main paper as new part of **Fig. 7** (as new panel C).

- Figure S20 does not make sense. First, the western blot in panel A was apparently generated by probing with both anti-gB and anti-GFP simultaneously with no way to distinguish the bands resulting from reactivity with each antibody. For example, there is no way to tell whether the band at about 37k is actually VP26-GFP or a degradation product of the 100k band. Similarly, there is no way to know whether the upper band actually represents reactivity with the putative anti-gB or cross reactivity of the anti-GFP antibody with some other BoHV protein. I cannot understand why the experiment was done in this way. Panels B and C also do not make sense. The box that the authors cut clearly contains the major capsid protein, VP5 (the most abundant protein by copy number in any herpesvirus virion), but they state that gB is the most abundant protein by peptide representation. This is odd to begin with, but their graph appears to show substantially greater abundances for peptides of VP11/12 and 13/14. It is possible that I have mis-read the graph because the authors do not indicate what is on the Y axis (what does “area” mean in this context?). The bottom line is that the figure does not provide evidence that their antibody reacts specifically with gB.
  The specificity of this antibody is cast into further doubt by the confocal images shown in Figure 8. They are of too low a resolution to tell for sure (and the lack of a separated red channel does not help), but the putative gB signal appears to be distributed evenly throughout the cytoplasm rather than localized on any membrane structures as would be expected for an envelope glycoprotein.
  The bottom line here is that I am unconvinced that this antibody is specifically directed against gB. Demonstration of reactivity in cells that express only gB is necessary to clarify this.

**Response:** Please accept our profound apology for the confusions we have caused. The Western Blot shown in the original Figure S20A in our first revision (panel 2 in **Figure 1** here) was indeed done using the anti-BoHV1 and anti-GFP antibodies simultaneously (both raised in mouse). It was taken from an early experiment (full results shown in Figure 1 here) when we were trying to identify the target band for the antibody obtained from the Friedrich Loeffler Institute (FLI). For this experiment, we used concentrated supernatant viruses produced by the WT and KO cells along with two controls (SFM and PR8). We then did Coomassie staining (panel 1), and two separate western blots one with both the anti- GFP antibody and the FLI antibody (panel 2), and the other with just the anti-GFP antibody (panel 3). By comparing the results from panel 2 to panel 3, it is clear that the FLI antibody targeted a viral protein that sat around 100kD, and that the GFP antibody is very specific to the VP26-GFP protein (41kD). Also, to avoid future confusion, we have now replaced the original Fig. S20A with a western blot done with the two primary antibodies (FLI antibody and anti-GFP antibody) applied sequentially, using two different secondary antibodies (Licor DyLight 800 for the FLI antibody and 680 for the GFP antibody) to separate the signals and to label VP26-GFP with a different color (presented as the new **Fig. S19A** on page 17 of the tracked “supplementary_data” document). This was how most of our westerns were conducted for the analyses.

Figure 1. Concentrated virus samples stained by Coomassie (panel 1), the FLI antibody against an unknown “envelope” protein of BoHV-1 with the anti-GFP antibody against VP26-GFP (panel 2), or the anti-GFP antibody against VP26-GFP only (panel 3).

Because we had limited records available except that the FLI antibody targeted an envelope protein of BoHV-1, it led us to believe that the target was gB, based on our initial western blot and Mass Spec analyses. As requested by the reviewer, to be sure of the antigen, we first cloned the ORFs of all the most likely viral envelope proteins, gB, gC, gD, gE, gG, gH, based on the genomic sequence for BoHV-1 strain cooper (ncbi genome ID KU198480.1). After expressing them in HEK293FT cells individually and western blot analyses, we detected no positive binding between the FLI antibody and any of these envelope proteins, proving the Reviewer right that indeed gB was not the protein being targeted by the FLI antibody (one out of three repeats shown as a new **Fig. S19D** on page 17 of the tracked “supplementary_data” file copied here as **Figure 2** shown above). We thus searched further and cloned one more viral protein, i.e. VP8 of BoHV-1 because it was the most abundant viral protein detected with the right size (~93kD). By doing this, we confirmed that VP8, a tegument protein of BoHV-1, is what the FLI antibody has been reacting with. Our experiments measured the abundance of VP8 in our viruses and infected cells, not any envelope protein. We have since revised our manuscript accordingly. Please see the representative western blot results copied here which shows that VP8 is the target of this antibody. As you can see in both western blots, the GFP antibody (JL-8 from Takara Bio) and the FLI antibody are very specific and do not cross react with each other’s target, and this has been consistent across our experiments. Please also see our revised Figure S19 on page 16-17 in our revised “supplementary data” file for more details on this.

Figure 2. Representative Western blot results on lysates harvested from HEK293FT transfected with plasmids overexpressing glycoproteins or VP8 of BoHV-1. The same membrane was hybridized with the primary anti-Flag antibody (rabbit) and the FLI antibody (mouse) together followed by secondary antibodies labelling the anti-Flag (Licor Donkey anti-rabbit 800) or the FLI antibody (Licor Donkey anti-mouse 680). -ve: empty vector, MOCK: lysate from supernatant of MOCK infected cells; BoHV-1: lysate from concentrated supernatant of cells infected with the GFP tagged BoHV-1.

**Part II – Major Issues: Key Experiments Required for Acceptance**
Please use this section to detail the key new experiments or modifications of existing experiments that should be absolutely required to validate study conclusions.

Generally, there should be no more than 3 such required experiments or major modifications for a "Major Revision" recommendation. If more than 3 experiments are necessary to validate the study conclusions, then you are encouraged to recommend "Reject".

- Reviewer #2: (No Response)
- Reviewer #3: Demonstration of their putative anti-gB antibody reactivity in cells that express only gB is necessary to show antibody specificity.

**Response:** Please see the response we have made on the same issue on lines 102-130 in this document.

**Part III – Minor Issues: Editorial and Data Presentation Modifications**
Please use this section for editorial suggestions as well as relatively minor modifications of existing data that would enhance clarity.

- Reviewer #2: I realize that Figure S20 is the 20th supplementary figure, but I cannot figure it out.
  “Western blot using anti BoHV1 Ab and an anti-GFP Ab against concentrated virus samples from TC supt of WT or 51KO cells. Black arrows point to band that matches VP26-GFP.”
  This antibody was used to stain lysates of viruses harvested from media “alongside” an anti-GFP antibody.
  I don’t understand – is this a GFP blot or a VP26 blot? Or both? If both, I only see one red VP26 band.
  Is red anti-GFP or anti-gB? Why would they use both Abs unless you could see them both- w two different colors? Is it supposed to be green as well?

**Response:** Please see our response to a similar comment from lines 75-101 in this document.

- In Fig S20C, there is a color for WT and one for MOCK. But the bars in the graph all look black. I am assuming that the gB, UL47, and gE , which seem to have 2 bars associated with them, the one to the right is gray.

**Response:** I have since added colour to the MOCK to visually separate the two. Please also see the response below on lines 176-181 for further clarification. Basically, due the way the samples were loaded and processed, there was slight cross contamination between the WT and MOCK samples, albeit at very low levels (<5%).

- Not clear why MOCK infected cells have viral proteins in them. Is this contamination from neighboring lanes? If so, please explain. Are all those proteins found by MS predicted to be in the 98 kDa range? If not, please discuss. If so, please discuss - just so that the figure is understandable. Confusing. Is the Coomassie stained band at 95 kDa supposed to be gB? Or, was that black box cut out and gB and all the other proteins detected by MS?

**Response:**

Thank you for pointing this out. The positive signals for some of the viral proteins in the MOCK samples are most likely due to cross-contamination as the MOCK samples were loaded immediately next to the WT infected samples. In addition, the same blade was used to cut out the gel pieces. Nevertheless, these MOCK samples should be true MOCK for the following reasons: a. our western blotting did not detect any positive viral protein signal, b. the virus titers from the MOCK samples were 0 pfu/ml, and c. the viral protein levels were less than 5% of that in the infected samples.

We also took the opportunity to replot this series of data by combining the abundance of VP13/14 and UL47, as they represent the same protein VP8 in BoHV-1 (new **Fig. S19C** on pages 16-17 of the supplementary data file). VP13/14 is an analogue in HSV-1 and in the protein database Uniprot where we obtained the peptide sequences for our Mass Spec analyses, the protein product of UL47 was listed as either VP13/14, UL47, or VP8. In addition, we corrected an error we made while calculating the abundance of VP8 in the MOCK samples. To our knowledge, there is no other mistake we have made regarding this set of data.

As for the MS experiment presented in Figure S19 B,C, we resolved the concentrated viral samples by SDS PAGE and stained the gel with Coomassie. Following the staining, the gel pieces spanning around 90-110kD that happened to overlap the darkest bands on the gel with matching sizes to the band detected by Western Blotting were cut out and sent for Mass Spec (S19A). Figure C showed all the proteins detected within those four gel pieces (two for samples collected from infected WT cells, two for MOCK samples).

Reviewer #3: (No Response)
